# Optimization of the multivariate polynomial public key for quantum safe digital signature

**DOI:** 10.1038/s41598-023-32461-3

**Published:** 2023-04-19

**Authors:** Randy Kuang, Maria Perepechaenko

**Affiliations:** Quantropi Inc, Ottawa, ON K1Z 8P8 Canada

**Keywords:** Engineering, Mathematics and computing

## Abstract

Kuang, Perepechaenko, and Barbeau recently proposed a novel quantum-safe digital signature algorithm called Multivariate Polynomial Public Key or MPPK/DS. The key construction originated with two univariate polynomials and one base multivariate polynomial defined over a ring. The variable in the univariate polynomials represents a plain message. All but one variable in the multivariate polynomial refer to noise used to obscure private information. These polynomials are then used to produce two multivariate product polynomials, while excluding the constant term and highest order term with respect to the message variable. The excluded terms are used to create two noise functions. Then four produced polynomials, masked with two randomly chosen even numbers over the ring, form the Public Key. The two univariate polynomials and two randomly chosen numbers, behaving as an encryption key to obscure public polynomials, form the Private Key. The verification equation is derived from multiplying all of the original polynomials together. MPPK/DS uses a special safe prime to prevent private key recovery attacks over the ring, forcing adversaries to solve for private values over a sub-prime field and lift the solutions to the original ring. Lifting entire solutions from the sub-prime field to the ring is designed to be difficult based on security requirements. This paper intends to optimize MPPK/DS to reduce the signature size by a fifth. We added extra two private elements to further increase the complexity of the private key recovery attack. However, we show in our newly identified optimal attack that these extra private elements do not have any effect on the complexity of the private recovery attack due to the intrinsic feature of MPPK/DS. The optimal key-recovery attack reduces to a Modular Diophantine Equation Problem or MDEP with more than one unknown variables for a single equation. MDEP is a well-known NP-complete problem, producing a set with many equally-likely solutions, so the attacker would have to make a decision to choose the correct solution from the entire list. By purposely choosing the field size and the order of the univariate polynomials, we can achieve the desired security level. We also identified a new deterministic attack on the coefficients of two univariate private polynomials using intercepted signatures, which forms a overdetermined set of homogeneous cubic equations. To the best of our knowledge, the solution to such a problem is to brute force search all unknown variables and verify the obtained solutions. With those optimizations, MPPK/DS can offer extra security of 384 bit entropy at 128 bit field with a public key size being 256 bytes and signature size 128 or 256 bytes using SHA256 or SHA512 as the hash function respectively.

## Introduction

Kuang, Perepechaenko, and Barbeau recently proposed a novel post-quantum cryptographic algorithm for key encapsulation based on multivariate polynomial multiplication for the key construction and division for the secret extraction^1^. This mechanism is called Multivariate Polynomial Public Key or MPPK. The novel MPPK mechanism gives rise to a key encapsulation mechanism called MPPK KEM and a digital signature scheme called MPPK/DS.

MPPK KEM evolved from a simple but elegant univariate polynomial scheme proposed by Kuang in 2021^2^ based on the following equation $\frac{B(x)f(x)}{B(x)h(x)} = \frac{f(x)}{h(x)}$ over a prime finite field $\mathbb {F}_p$. Here, the common base univariate polynomial *B*(*x*) of a high order is used as a noise injector to the scheme, and two univariate solvable polynomials *f*(*x*) and *h*(*x*) are used to transmit the secret message. The product polynomials ${\mathscr {P}}(x) = B(x) f(x)$ and ${\mathscr {Q}}(x) = B(x) h(x)$, considered without their constant terms, are used to establish the public key. This public key construction offers non-deterministic private key recovery. However, the secret recovery attack would extract the secret with polynomial time complexity under the assumption of the Generalized Riemann Hypothesis or GRH used by Evdokimov in 1994^3^. To overcome this vulnerability, Kuang and Barbeau proposed to use multivariate base polynomial $B(x_0, x_1, \dots , x_m)$, where $x_0$ denotes the message and $x_1, \dots , x_m$ denote noise variables over $\mathbb {F}_p$^4,5^. Then the same key construction algorithm was used to create two public multivariate polynomials $\Phi (x_0, x_1, \dots , x_m)$ from $f(x_0)B(x_0, x_1, \dots , x_m)$ and $\Psi (x_0, x_1, \dots , x_m)$ from $h(x_0)B(x_0, x_1, \dots , x_m)$ by excluding the constant and highest order terms with respect to the message variable $x_0$ from the product polynomials. The excluded terms with respect to $x_0$ are used to create noise functions, which are encrypted over the same prime field $\mathbb {F}_p$. This enhanced scheme improves the security of the secret, however, the private key becomes vulnerable. To overcome this vulnerability, Kuang, Perepechaenko, and Barbeau^1^ applied the noise function encryption over a hidden ring $\mathbb {Z}/S\mathbb {Z}$, increasing the security of the private key and maintaining the security of the secret.

Another scheme based on the MPPK mechanism, called MPPK digital signature or MPPK/DS, was recently proposed by Kuang, Perepechaenko, and Barbeau^6^. As opposed to MPPK KEM key pair construction over the prime field $\mathbb {F}_p$, MPPK/DS chooses the associated ring $\mathbb {Z}/{\phi (p)}\mathbb {Z}$ to construct its public key in a similar way as in MPPK KEM. The major difference in the public key construction mechanism is the obscurity of the public key polynomials $\Phi (x_0, x_1, \dots , x_m)$ with even integer $R_0$ and $\Psi (x_0, x_1, \dots , x_m)$ with even value $R_n$ over $\mathbb {Z}/{\phi (p)}\mathbb {Z}$. In MPPK/DS, the prime number *p* is selected to be a generalized safe prime of the form $p=2^x q + 1$. The verification equation is derived from the equation $f(x_0)[h(x_0)B(x_0, x_1, \dots , x_m)] = h(x_0)[f(x_0)B(x_0, x_1, \dots , x_m)]$ $\text {mod } \phi (p)$, by taking modular exponentiation with a randomly selected secret base *g*. MPPK/DS offers relatively small key sizes and signature sizes and outperforms all NIST finalists^7^.

In this paper, we propose an optimization of the MPPK/DS^6^ to further reduce the key pair sizes and signature size, as well as strengthen its security. We also discuss the potential side-channel-resistant implementation of the optimized MPPK signature algorithm.

### Related work

National Institute of Standards and Technology (NIST) started the standardization process of Post-Quantum Cryptography (PQC) in 2017^8^ with 69 candidates. The first round ended in 2019 with 26 candidates entering into the second round^9^. Only four candidates for KEM: code-based Classic McEliece^10^ and lattice-based CRYSTALS-KYBER^11^, NTRU^12^, and SABER, as well as three candidates for digital signatures: lattice-based CRYSTALS-DILITHIUM^13^ and FALCON^14^, and multivariate Rainbow^15^ entered into the third round in 2021^16^. Kyber became the only KEM candidate for standardization as announced by NIST in^17^, and CRYSTALS-Dilithium, FALCON, and SPHINCS+^18^ are the three digital signature candidates for standardization. Following this announcement, NIST issues a new call for digital signatures and emphasized that the primary interest is in general-purpose signature schemes that are not based on structured lattices.

Some vulnerabilities of NIST round 3 finalists were reported in early 2022. Damien Robert in 2022 first reported an attack on the Supersingular Isogeny Diffie-Hellman or SIDH^19^ in polynomial time^20^, and later Castryck and Decru reported their more efficient key recovery attack on SIDH^21^, achieving key recovery for NIST security level V in less than 2 h with a laptop. A new cryptoanalysis was recently proposed by Wenger et al. in 2022^22^, using Machine Learning or ML for secret recovery of the lattice-based schemes. Their proposed attack can fully recover secrets for small-to-midsize LWE instances with sparse binary secrets, up to lattice dimensions of $d= 128$, and may scale to attack real-world LWE-based cryptosystems. Attacking lattice-based schemes with ML transformers seems to be a promising area, thus, the team is working on further advancing the capability of their attack to target larger parameter sets. However, it is still unclear of the amount of time and resources needed to achieve this goal. Nevertheless, this attack opened the door to an entirely new era of cryptoanalysis using ML, especially when combining ML with quantum computing. Among the digital signature schemes Rainbow scheme, based on a multivariate public key cryptosystem, had a reported attack. Ward Beullens in early 2022 reported an attack on Rainbow that uses a standard laptop and requires an average of 53 h^23^. These recent attacks on well-explored PQC algorithms indicate that further exploration of novel PQC algorithms for both KEM and Digital signature are highly necessary.

On the other hand, Gottesman and Chuang in 2001 proposed a scheme of quantum digital signature or QDS by importing the ideas of classical public key cryptography into the quantum world based on their proposed quantum one-way function^24^. After then, several explorations and principal implementations have been reported by Admiri et al. in 2016^25^, Yin et al. in 2016^26^, Roberts et al. in 2017^27^, Yin et al. in 2017^28,29^, Zhao et al. in 2021^30,31^, Lu et al. in 2021^32^. Although QDS offers the information theoretical secure digital signature, Gottesman and Chuang^24^ have pointed out its disadvantages such as impossible to sign a general unknown quantum state, limited copies of the public key to be shared with recipients, especially its limitation of applicability over today’s internet. In addition to the mentioned limitations, the signature performance using QDS may be another limitation. The reported signature rates are varied such as the recorded breakthrough rate 0.98 s/bit at 103 km from Ding et al. in 2020^33^ and 14.9 s/bit efficient QDS without symmetrization step from Lu et al.^32^. In general, QDS signature performance is about six orders of magnitude smaller than QPC digital signature performance.

In contrast to QDS for quantum secure digital signature with some limitations as discussed in the above, PQC digital signature schemes would be much more applicable for Quantum Key Distribution or QKD. It is well-known that QKD requires a classical channel for the post-processing, requiring trusted authentication to avoid the Man-in-The-Middle or MITM attack. With PQC digital signature, QKD could eliminate the pre-shared secret which is always the weak point for QKD. QDS may be able to apply for short distance QKD, but for a long distance QKD such as Twin-Field QKD over 830 km by Wang et al. in 2022^34^, QKD network by Fan-Yuan et al. in 2021^35^ and in 2022^36^, quantum safe digital signature would make the entire communication networks be quantum safe without the pre-shared secret.

### Contribution

In this work, we proposed an optimized version of the MPPK/DS algorithm^6^. The following changes were made to the original MPPK/DS scheme:

1. Functions $E_{\phi }(x_0)$ and $E_{\psi }$ are chosen to be equal to $\Phi (x_0, x_1=0, \dots , x_m=0)$ and $\Psi (x_0, x_1=0, \dots , x_m=0)$ respectively. This change is done to remove the terms associated with single variable $x_0$ and reduce the public key by *m* elements chosen over $\mathbb {Z}/\varphi (p)\mathbb {Z}$;
2. Two new private randomly chosen numbers $\alpha , \beta \in \mathbb {Z}/\varphi (p)\mathbb {Z}$ are introduced. The values $\alpha , \beta$ are chosen with conditions $GCD(\alpha , \varphi (p)) = 1$ and $GCD(\beta , \varphi (p)) = 1$. These values are used to mask the public key polynomials $\Phi (\cdot )$ and $\Psi (\cdot )$. That is, public key elements are obscured using different values as follows: $R_0$ is used for ${\mathscr {N}}_0(\cdot )$, $R_n$ for ${\mathscr {N}}_n(\cdot )$, $\alpha R_0$ for $\Phi (\cdot )$, and $\beta R_n$ for $\Psi (\cdot )$;
3. The private key is organized into four univariate polynomials of order $\lambda$: $a(x_0), b(x_0), c(x_0), d(x_0)$;
4. A side-channel resistant implementation of the signing algorithm with $a(x_0), b(x_0), c(x_0)$, and $d(x_0)$ was proposed.

We have conducted a security analysis in “Security analysis”, and discovered an efficient spoofing attack with classical complexity ${\mathscr {O}}((m \ log_2 p)^{12} p^4)$, compared to the ${\mathscr {O}}( p^{4+m})$ in the original MPPK/DS security analysis.

## MPPK/DS optimization

In MPPK/DS^1^, the security parameters are the generalized safe prime $p=2^xq+1$, with $x \in \mathbb {Z}^{+}$ and an odd prime *q*, a positive integer *n*, representing the order of message variable $x_0$ in the base multivariate polynomial ${\mathscr {B}}(x_0, x_1, \dots , x_m)$, and a positive integer $\lambda$ representing the order of univariate polynomials $f(x_0)$ and $h(x_0)$. Variable $x_0$ is associated with the secret, while variables $x_1, \dots , x_m$ represent noise. The noise variables in the base multivariate polynomial ${\mathscr {B}}(x_0, x_1, \dots , x_m)$ enable the signature verifier to have the freedom to generate various values of the public key polynomials ${\mathscr {P}}(\cdot ), {\mathscr {Q}}(\cdot )$ as well as noise functions ${\mathscr {N}}_0(\cdot )$ and ${\mathscr {N}}_{m}(\cdot )$ for the same secret $x_0$ but different noise values. We generally maintain these definitions of security parameters in this paper.

In this section, we will first briefly describe the MPPK DS, followed by the key construction with the above optimization considerations, next derive the signature verification equation, then establish the signing algorithm and finally discuss the verification algorithm. For the remainder of this work, we replace $\varphi (p)$ with $p-1$ and $\mathbb {Z}/\varphi (p)\mathbb {Z}$ with $\mathbb {Z}/(p-1)\mathbb {Z}.$

### The motivation of MPPK

The fundamental idea of multivariate polynomial public key or MPPK is rooted to a simple algebra equation

1 $$\begin{aligned} \frac{{\mathscr {B}}(x_0, x_1, \dots , x_m)f(x_0)}{{\mathscr {B}}(x_0, x_1, \dots , x_m)h(x_0)} = \frac{{\mathscr {P}}(x_0, x_1, \dots , x_m)}{{\mathscr {Q}}(x_0, x_1, \dots , x_m)} = \frac{f(x_0)}{h(x_0)} \end{aligned}$$

with two univariate polynomial $f(x_0)$, $h(x_0)$ and a common multivariate polynomial ${\mathscr {B}}(x_0, x_1, \dots , x_m)$. Equation (1) demonstrates following very interesting characteristics:

- the division is invariant from any ${\mathscr {B}}(x_0, x_1, \dots , x_m)$, regardless its specific expression and the number of variables $x_1, \dots , x_m$. It is always dictated by $f(x_0)$ and $h(x_0)$.
- it implies that we can establish a new public key algorithm with the private key to be $f(x_0)$ and $h(x_0)$ and the public key to be ${\mathscr {P}}(x_0, x_1, \dots , x_m)$ and ${\mathscr {Q}}(x_0, x_1, \dots , x_m)$.
- it is naturally to consider the variable $x_0$ as the message variable for the secret exchange and variables $x_1, \dots , x_m$ as noise variables.

Based on Eq. (1), Kuang et al. in 2022^1^ proposed a key encapsulation mechanism or MPPK KEM. Kuang and Perepechaenko further proposed a new homomorphic MPPK or HPPK Kem by introducing homomorphic encryption on the plain public polynomials ${\mathscr {P}}(x_0, x_1, \dots , x_m)$ and ${\mathscr {Q}}(x_0, x_1, \dots , x_m)$ over a hidden ring^37^.

On the other hand, Eq. (1) can be rewritten with a cross-multiplication form as

2 $$\begin{aligned} {\mathscr {Q}}(x_0, x_1, \dots , x_m)f(x_0) = {\mathscr {P}}(x_0, x_1, \dots , x_m)h(x_0), \end{aligned}$$

which can be used to develop a signature verification equation by leveraging a fact: if $a = b \bmod {\varphi (p)}$, then for any $g\ne 0, 1 \in \mathbb {F}_p$, $g^a = g^b \bmod {p}$. That is the central idea of MPPK DS. Certain techniques must be applied to protect the private key from the public key and the signature attacks. Some key features from MPPK DS are

- small sizes of private key, public key and signature, generally smaller than RSA-2048.
- better performance for key generation, signing, and verifying, comparing with NIST DS candidates.
- generic for any devices from ARM to X86.
- randomized signing with a base *g* per signing and randomized verifying with noise variables.

### Key construction

Unless stated otherwise, all the arithmetic during the key generation procedure is performed modulo $p-1$. Using the same definitions as in the MPPK/DS^6^, we randomly choose three polynomials with over the ring $\mathbb {Z}/(p-1)\mathbb {Z}$, a base multivariate polynomial of order *n*

3 $$\begin{aligned} {\mathscr {B}}(x_0, x_1, \dots , x_m) = \sum _{j=1}^{m} \sum _{i=0}^{n} c_{ij} x_j x_0^i = {\mathscr {B}}_1(x_0)x_1 + \dots + {\mathscr {B}}_m(x_0) x_m \end{aligned}$$

with ${\mathscr {B}}_j(x_0) =\sum _{i=0}^nx_0^i$, and two univariate polynomials of order $\lambda$

4 $$\begin{aligned} f(x_0) = f_0 + f_1 x_0 + \dots + f_{\lambda } x_0^{\lambda } \ \text { and } \ h(x_0) = h_0 + h_1 x_0 + \dots + h_{\lambda } x_0^{\lambda } \end{aligned}$$

We have simplified the monomials in MPPK/DS^6^, and consider only the monomials of the form $x_0^{i}x_j$ as in Eq. (3). The base polynomial can be considered as a linear multivariate polynomial with coefficients being univariate polynomials ${\mathscr {B}}_j(x_0)$ for all $j=1, \dots , m$.

Two product polynomials $\phi (\cdot )$ and $\psi (\cdot )$ are then constructed as

5 $$\begin{aligned} \phi (x_0, x_1, \dots , x_m) = {\mathscr {B}}(x_0, x_1, \dots , x_m) f(x_0) = \sum _{j=1}^{m} \left( \sum _{k=0}^{n+\lambda } \phi _{kj}x_0^i\right) x_j \end{aligned}$$

and

6 $$\begin{aligned} \psi (x_0, x_1, \dots , x_m) = {\mathscr {B}}(x_0, x_1, \dots , x_m) h(x_0) = \sum _{j=1}^{m} \left( \sum _{k=0}^{n+\lambda } \psi _{kj}x_0^i\right) x_j \end{aligned}$$

with $\phi _{kj} = \sum _{s+t=k} f_s c_{tj}$ and $\psi _{kj} = \sum _{s+t=k} h_s c_{tj}$. Equation (5) can be rewritten as

7 $$\begin{aligned} \phi (x_0, x_1, \dots , x_m) = f_0{\mathscr {B}}_0(x_1, \dots , x_m) + \Phi (x_0, x_1, \dots , x_m) + f_{\lambda }{\mathscr {B}}_n(x_1, \dots , x_m)x_0^{n+\lambda } \end{aligned}$$

with

8 $$\begin{aligned} \Phi (x_0, x_1, \dots , x_m) =\sum _{j=1}^{m} \left( \sum _{k=1}^{n+\lambda -1} \phi _{kj}x_0^k\right) x_j, \end{aligned}$$

and Eq. (6) can be rewritten as

9 $$\begin{aligned} \phi (x_0, x_1, \dots , x_m) = f_0{\mathscr {B}}_0(x_1, \dots , x_m) + \Psi (x_0, x_1, \dots , x_m) + f_{\lambda }{\mathscr {B}}_n(x_1, \dots , x_m)x_0^{n+\lambda } \end{aligned}$$

with

10 $$\begin{aligned} \Psi (x_0, x_1, \dots , x_m) =\sum _{j=1}^{m} \left( \sum _{k=1}^{n+\lambda -1} \phi _{kj}x_0^k\right) x_j \end{aligned}$$

We refer to the multivariate product polynomials $\phi (x_0, x_1, \dots , x_m)$ and $\psi (x_0, x_1, \dots , x_m)$ as plain product polynomials. They can not be directly used as public key as there exists a polynomial time factorization algorithm on univariate polynomials^3^. In order to protect the plain product polynomials, we consider their components separately. First, we mask the components ${\mathscr {B}}_0(\cdot )$ and ${\mathscr {B}}_n(\cdot )$ with randomly chosen even numbers $R_0, R_n \in \mathbb {Z}/(p-1)\mathbb {Z}$ respectively, producing two functions

11 $$\begin{aligned} {\mathscr {N}}_0(x_1, \dots , x_m) = R_0 {\mathscr {B}}_0(x_1, \dots , x_m) \ \text {mod p-1} = (R_0 c_{01} x_1 + \dots + R_0 c_{0m} x_m ) \ \text {mod (p-1)} \end{aligned}$$

and

12 $$\begin{aligned} {\mathscr {N}}_n(x_0, x_1, \dots , x_m) = R_n {\mathscr {B}}_n(x_1, \dots , x_m) x_0^{n+\lambda }\ \text {mod (p-1)} = (R_n c_{n1} x_1 + \dots + R_n c_{nm} x_m ) x_0^{n+\lambda } \ \text {mod (p-1)}. \end{aligned}$$

The functions ${\mathscr {N}}_0(x_1, \dots , x_m)$ and ${\mathscr {N}}_n(x_0, x_1, \dots , x_m)$ are called noise functions in MPPK/DS^6^. Then we randomly choose two number $\alpha$ and $\beta$ from $\mathbb {Z}_{p-1}$ such that $GCD(\alpha , p-1) = 1$ and $GCD(\beta , p-1) = 1$, to obscure $\Phi (\cdot )$ and $\Psi (\cdot )$ as

13 $$\begin{aligned} {\mathscr {P}}(x_0, x_1, \dots , x_m) = (\alpha R_0 \Phi (x_0, x_1, \dots , x_m) ) \ \text {mod (p-1)} = \sum _{j=1}^{m} \left[ \sum _{k=1}^{n+\lambda -1} (\alpha R_0 \phi _{kj} \ \text {mod (p-1)} \right] x_0^k) x_j = \sum _{j=1}^{m} p_j(x_0) x_j, \end{aligned}$$

with $$p_j(x_0) = \sum _{k=1}^{n+\lambda -1} (\alpha R_0 \phi _{kj} \ \text {mod (p-1)}) x_0^k$$,

14 $$\begin{aligned} {\mathscr {Q}}(x_0, x_1, \dots , x_m) = (\beta R_n \Psi (x_0, x_1, \dots , x_m) ) \ \text {mod (p-1)} = \sum _{j=1}^{m} \left[ \sum _{k=1}^{n+\lambda -1} (\beta R_n \psi _{kj} \ \text {mod (p-1)} \right] x_0^k) x_j =\sum _{j=1}^{m} q_j(x_0) x_j, \end{aligned}$$

with $q_j(x_0) =\sum _{k=1}^{n+\lambda -1} (\beta R_n \psi _{kj} \ \text {mod (p-1)})$. We refer to the polynomials ${\mathscr {P}}(\cdot ), {\mathscr {Q}}(\cdot )$ as well as noise functions ${\mathscr {N}}_0(\cdot ), {\mathscr {N}}_n(\cdot )$ as modular multiplicatively encrypted polynomials. These polynomials form the public key:

- ${\mathscr {N}}_0(x_1, \dots , x_m)$, which we denote ${\mathscr {N}}_0[m]$
- ${\mathscr {N}}_n(x_0, x_1, \dots , x_m)$, which we denote ${\mathscr {N}}_n[m]$
- ${\mathscr {P}}(x_0, x_1, \dots , x_m)$, which we denote ${\mathscr {P}}[(n+\lambda -1)\times m]$
- ${\mathscr {Q}}(x_0, x_1, \dots , x_m)$, which we denote ${\mathscr {Q}}[(n+\lambda -1)\times m]$.

An attentive reader will notice that Eqs. (13) and (14) are essentially the same as in MPPK/DS in^6^ except for the multiplication by the values $\alpha , \beta$, and every term is associated with a noise variable. The private key of the optimized MPPK/DS consists of

- $$f(x_0)$$ and $$h(x_0)$$
- $$R_0, R_n, \alpha , \beta$$

### Derivation of the verification equation

We start from the following equation modulo $p-1$

15 $$\begin{aligned} f(x_0)[h(x_0){\mathscr {B}}(x_0, x_1, \dots , x_m)] = h(x_0)[f(x_0){\mathscr {B}}(x_0, x_1, \dots , x_m)], \text { or equivalently } f(x_0) \psi (x_0, x_1, \dots , x_m) = h(x_0) \phi (x_0, x_1, \dots , x_m). \end{aligned}$$

Multiplying Eq. (15) by $R_0R_n$ on both sides and using Eq. (5) to (14), we can derive the following expression

16 $$\begin{aligned} a(x_0) {\mathscr {Q}}(x_0, x_1, \dots , x_m) = b(x_0) {\mathscr {P}}(x_0, x_1, \dots , x_m) + c(x_0) {\mathscr {N}}_0(x_1, \dots , x_m) + d(x_0) {\mathscr {N}}_n(x_0, x_1, \dots , x_m) \ \text {mod (p-1)}, \end{aligned}$$

where

17 $$\begin{aligned} {\left\{ \begin{array}{ll} a(x_0) = \frac{R_0}{\beta } f(x_0) \ \text {mod (p}-1)= a_0 + a_1 x_0 + \dots + a_{\lambda }x_0^{\lambda }\\ b(x_0) = \frac{R_n}{\alpha } h(x_0) \ \text {mod (p}-1)= b_0 + b_1 x_0 + \dots + b_{\lambda }x_0^{\lambda }\\ c(x_0) = R_n[h(x_0)f_0 - f(x_0)h_0] \ \text {mod (p}-1) = c_1 x_0 + \dots + c_{\lambda }x_0^{\lambda } \\ d(x_0) = R_0[h(x_0)f_{\lambda } - f(x_0)h_{\lambda }] \ \text {mod (p-1)}= d_0 + d_1 x_0 + \dots + d_{\lambda -1}x_0^{\lambda -1}. \end{array}\right. } \end{aligned}$$

We now randomly choose a base $g \ne 0,1 \in \mathbb {F}_p$, and take the base *g* to the power of expression in Eq. (16) as follows

18 $$\begin{aligned} ( g^{a(x_0)})^{ {\mathscr {Q}}(x_0, x_1, \dots , x_m)} = (g^{b(x_0)})^{{\mathscr {P}}(x_0, x_1, \dots , x_m)} (g^{c(x_0)})^{{\mathscr {N}}_0(x_1, \dots , x_m)} (g^{c(x_0)})^{{\mathscr {N}}_n(x_0, x_1, \dots , x_m)} \ \text {mod p}. \end{aligned}$$

Then we define the signature to be comprised of the following elements

19 $$\begin{aligned} {\left\{ \begin{array}{ll} A = g^{a(x_0)} \ \text {mod p} \\ B = g^{b(x_0)} \ \text {mod p} \\ C = g^{c(x_0)} \ \text {mod p} \\ D = g^{d(x_0)} \ \text {mod p}. \end{array}\right. } \end{aligned}$$

Using Eq. (19), we derive the signature verification equation from Eq. (18) as follows

20 $$\begin{aligned} A^{ {\mathscr {Q}}(x_0, x_1, \dots , x_m)} = B^{{\mathscr {P}}(x_0, x_1, \dots , x_m)} C^{{\mathscr {N}}_0(x_1, \dots , x_m)} D^{{\mathscr {N}}_n(x_0, x_1, \dots , x_m)} \ \text {mod p}. \end{aligned}$$

Although we can use original private polynomials $f(x_0)$ and $h(x_0)$, together with private secret values $R_0, R_n, \alpha , \beta$ as the private key, it is far better to use the derived univariate polynomials in Eq. (17) as the private key to avoid potential side-channel attacks on polynomial evaluations^38^. Now we form the key pair for the optimized MPPK/DS:

**Public Key:** $${\mathscr {N}}_0[m], {\mathscr {N}}_n[m], {\mathscr {P}}[(n+\lambda -1)\times m] , {\mathscr {Q}}[(n+\lambda -1)\times m]$$
**Private Key:** $$a[\lambda +1], b[\lambda +1], c[\lambda ], d[\lambda ].$$

The total public key size is then calculated as $2m(n+\lambda )$ elements of $\mathbb {Z}/(p-1)\mathbb {Z}$ and private key size as $4\lambda +2$ elements of $\mathbb {Z}/(p-1)\mathbb {Z}$. The signature sizes are $4\times L$ with L to be the length of the signing message. It is clear that optimized MPPK/DS reduces the signature size from 5 to 4 elements of $\mathbb {F}_p$.

### Signing with optimized MPPK/DS

Signing with optimized MPPK/DS is a straightforward three-step process:

1. Generate the hash code with given message/document *m*: $x_0 = HASH(m)$, and if the hash returns with a length $|x_0|_2$ larger than field length $log_2 p$, then segment it into segments $x_0[i]$ over $\mathbb {Z}/(p-1)\mathbb {Z}$. Perform steps 2 and 3 for each segment $x_0[i]$ and concatenate them together to form the signature tuple.
2. Evaluate $$\bar{a}= a(x_0) \ \text {mod (p-1)}, \bar{b}= b(x_0) \ \text {mod (p-1)}, \bar{c}= c(x_0) \ \text {mod (p-1)}, \bar{d}= d(x_0) \ \text {mod (p-1)}$$.
3. Randomly choose a base *g* from $\mathbb {F}_p$ and evaluate $A = g^{\bar{a}} \ \text {mod p}, B = g^{\bar{b}} \ \text {mod p}, C = g^{\bar{c}} \ \text {mod p}, D = g^{\bar{d}} \ \text {mod p}$. Note that *g* is chosen differently for every message *m*.

The tuple $S = \{A, B, C, D\}$ forms the signature for the message/document *m*. With the randomly chosen base $g \ne 0,1$, MPPK/DS naturally enables randomized signature, even for the same message *m*, repeated signing would produce a totally different signature.

### Verifying with optimized MPPK/DS

Verifying a signature $S = \{A, B, C, D\}$ signed by a true signer is also straightforward, using the verification equation Eq. (18). It, too, is a three-step process:

1. Generate the hash code with given message/document *m*: $x_0 = HASH(m)$, and if the hash returns with a length $|x_0|_2$ larger than field length $log_2 p$, then segment it into segments $x_0[i]$ over $\mathbb {Z}/(p-1)\mathbb {Z}$ and also segment each signature element into segments *S*[*i*]. Perform steps 2 and 3 for each segment $x_0[i]$ and *S*[*i*] for verification.
2. Randomly choose noise variable values $x_1, \dots , x_m$ from $\mathbb {Z}/(p-1)\mathbb {Z}$ and evaluate ${\mathscr {P}}(x_0, x_1, \dots , x_m) \ \text {mod (p-1)}, {\mathscr {Q}}(x_0, x_1, \dots , x_m)$ $\ \text {mod (p-1)}, {\mathscr {N}}_0(x_1, \dots , x_m) \ \text {mod (p-1)}, {\mathscr {N}}_n(x_0, x_1, \dots , x_m) \ \text {mod (p-1)}$
3. Verify if $A^{\bar{{\mathscr {Q}}}} = B^{\bar{{\mathscr {P}}}} C^{\bar{{\mathscr {N}}_0}} D^{\bar{{\mathscr {N}}_n}} \ \text {mod p}$ is true. If it is true, the verification is successful. The verification can be repeatedly performed as many times as the verifier wants with different choices of noise variables.

### Toy example

In this subsection, we use a toy example to demonstrate how the optimized MPPK/DS works. Suppose that the security parameters are chosen to be $p = 2^5 \times 11 + 1 = 353$, thus $\phi (p) = 352$, $n=\lambda = m = 2.$

**Key generation**

1. Three initial polynomials:
  $$f(x_0) = 269 + 111x_0 + 26x_0^2$$
  $$h(x_0) = 184 + 167x_0 + 167x_0^2$$
  $${\mathscr {B}}(x_0, x_1, x_2) =(100+296x_0 + 65x_0^2)x_1 + (210 + 36 x_0 + 68x_0^2) x_2$$ $${\mathscr {B}}_0(x_1, x_2) = 100x_1 +210x_2, \ {\mathscr {B}}_1(x_1, x_2) = 296x_1 +36x_1, \ {\mathscr {B}}_2(x_1, x_2) = 65x_1 + 68x_2$$
2. Two product polynomials:
  $$\phi (x_0, x_1, x_2)={\mathscr {B}}(x_0, x_1, x_2) f(x_0) = (148+260x_0 + 141x_0^2 + 127x_0^3 + 282x_0^4)x_1 + (170 + 258x_0 +292x_0^2+ 36x_0^3 +8x_0^4 )x_2$$ $$\Phi (x_0, x_1, x_2)= (260x_0 + 141x_0^2 + 127x_0^3 )x_1 + ( 258x_0 +292x_0^2+ 36x_0^3)x_2$$
  $$\psi (x_0, x_1, x_2)={\mathscr {B}}(x_0, x_1, x_2) h(x_0) =(96+60x_0 + 300x_0^2 +95x_0^3 + 295x_0^4)x_1 + (272 + 158x_0 +90x_0^2+ 120x_0^3 +92x_0^4 ) x_2$$ $$\Psi (x_0, x_1, x_2)= (60x_0 + 300x_0^2 +95x_0^3 )x_1 + ( 158x_0 +90x_0^2+ 120x_0^3 ) x_2$$
3. Encrypted or obscured product polynomials:
  $$R_0 = 182, R_n = 300, \alpha =\beta = 1$$
  $${\mathscr {P}}(x_0, x_1, x_2)= R_0 \alpha \Phi (x_0, x_1, x_2) \ \text {mod 352} = (152x_0 + 318x_0^2 + 234x_0^3 )x_1 + ( 140x_0 +344x_0^2+ 216x_0^3)x_2$$
  $${\mathscr {Q}}(x_0, x_1, x_2)= R_n\beta \Psi (x_0, x_1, x_2) \ \text {mod 352} = (48x_0 + 240x_0^2 + 340x_0^3 )x_1 + ( 232x_0 +248x_0^2+ 96x_0^3)x_2$$
  $${\mathscr {N}}_0( x_1, x_2)= R_0{\mathscr {B}}_0( x_1, x_2) \ \text {mod 352} = 248x_1 +204x_2$$
  $${\mathscr {N}}_n( x_1, x_2)= R_n{\mathscr {B}}_n( x_1, x_2)x_0^4 \ \text {mod 352} = (140x_1 +336x_2)x_0^4$$
4. Private polynomials:
  $$a(x_0) = \frac{R_0}{\beta } f(x_0) \ \text {mod 352} = 30 + 138x_0 + 156x_0^2$$
  $$b(x_0)= \frac{R_n}{\alpha } h(x_0) \ \text {mod 352} = 288 + 116x_0 + 116x_0^2$$
  $$c(x_0)= R_n[h(x_0)f_0 - f(x_0)h_0] \ \text {mod 352} = 292x_0 + 132x_0^2$$
  $$d( x_0)= R_0[h(x_0)f_{\lambda } - f(x_0)h_{\lambda }] \ \text {mod 352} = 110 + 190x_0$$

**Signing**

1. Evaluate private polynomials with $x_0=48$:
  $$a(x_0) = 30 + 138*48 + 156*48^2 = 350 \ \text {mod 352}$$
  $$b(x_0)= 288 + 116*48 + 116*48^2=320 \ \text {mod 352}$$
  $$c(x_0)= 292*48 + 132*48^2 = 288 \ \text {mod 352}$$
  $$d( x_0)= 110 + 190*48 =78 \ \text {mod 352}$$
2. Generate signature with randomly chosen base $g=277$
  $$A = 277^{a(48)} \ \text {mod 353} = 277^{350} \ \text {mod 353} = 262$$
  $$B = 277^{b(48)} \ \text {mod 353} =277^{320} \ \text {mod 353} = 187$$
  $$C = 277^{c(48)} \ \text {mod 353} =277^{288} \ \text {mod 353} = 22$$
  $$D = 277^{d(48)} \ \text {mod 353} =277^{78} \ \text {mod 353} =159$$

**Verifying**

1. Evaluate public polynomials with $x_0=48$:
  $$x_0 = 48, x_1 = 51, x_2=121$$
  $${\mathscr {P}}(x_0, x_1, x_2)= (152*48 + 318*48^2 + 234*48^3 )*51 + ( 140*48 +344*48^2+ 216*48^3)*121 = 32$$
  $${\mathscr {Q}}(x_0, x_1, x_2)= (48*48 + 240*48^2 + 340*48^3 )*51 + ( 232*48 +248*48^2+ 96*48^3)*121 = 320$$
  $${\mathscr {N}}_0( x_1, x_2)= 248*51 +204*121 = 20$$
  $${\mathscr {N}}_n( x_1, x_2)= (140*51 +336*121)*48^4=256$$
2. Verify signature
  $$A^{{\mathscr {Q}}(x_0, x_1, x_2)} \ \text {mod 353} = 262^{320} \ \text {mod 353} = {\textbf {337}}$$
  $$B^{{\mathscr {P}}(x_0, x_1, x_2)} C^{{\mathscr {N}}_0( x_1, x_2)} D^{{\mathscr {N}}_n( x_1, x_2)} = 187^{32}*22^{20}*159^{256} \ \text {mod 353} = {\textbf {337}}$$

**Re-verifying**

1. Evaluate public polynomials for $x_0=48$:
  $$x_0 = 48, x_1 = 259, x_2=324$$
  $${\mathscr {P}}(x_0, x_1, x_2)= (152*48 + 318*48^2 + 234*48^3 )*259 + ( 140*48 +344*48^2+ 216*48^3)*324 = 128$$
  $${\mathscr {Q}}(x_0, x_1, x_2)= (48*48 + 240*48^2 + 340*48^3 )*259 + ( 232*48 +248*48^2+ 96*48^3)*324 = 160$$
  $${\mathscr {N}}_0( x_1, x_2)= 248*259 +204*324 = 88$$
  $${\mathscr {N}}_n( x_1, x_2)= (140*259 +336*324)*48^4=256$$
2. Verify signature
  $$A^{{\mathscr {Q}}(x_0, x_1, x_2)} \ \text {mod 353} = 262^{160} \ \text {mod 353} = {\textbf {185}}$$
  $$B^{{\mathscr {P}}(x_0, x_1, x_2)} C^{{\mathscr {N}}_0( x_1, x_2)} D^{{\mathscr {N}}_n( x_1, x_2)} = 187^{128}*22^{88}*159^{256} \ \text {mod 353} = {\textbf {185}}$$

## Security analysis

To be considered quantum-safe, an algorithm or protocol must meet the following criteria:

- Resistance to known quantum attacks: The algorithm or protocol should be resistant to known quantum attacks. Fault-tolerant scalable quantum computers are capable of efficiently solving the integer factoring problem and the discrete logarithm problem, which form the security basis of most of the commonly used digital signatures schemes today.
- Security: The algorithm or protocol should provide the same level of security as existing digital signature schemes or higher. This means that it should be resistant to all known classical attacks, and that corresponding quantum security level still meets desired entropy requirements.

In this section, we present attacks on the optimized MPPK/DS scheme that we have discovered up to this date, and provide estimates of the complexity of these attacks. Any attack on MPPK/DS entails selective forgery of the signatures. In other words, the goal of the adversary is to generate a malicious signature that will pass the verification process. We discuss private key attacks, signature attacks, as well as direct spoofing attacks. These attacks and their corresponding complexities are summarized in “Security conclusion”.

### Private key recovery from public key

The adversary looking to use private key elements to generate a malicious signature that passes verification requires the knowledge of the following elements

$$\begin{aligned} \frac{R_0f_i}{\beta },\frac{R_nh_i}{\alpha }, h'_i=\beta h_i, \text { and } f'_i=\alpha f_i \ \forall i \in \{0, \dots , \lambda \}. \end{aligned}$$

Indeed, it suffices to find these elements since the signature component *A* can be expressed as

$$\begin{aligned} A = g^{a(x_0)} = g^{\frac{R_0}{\beta }f(x_0)} = \prod _{i = 0}^{\lambda }(g^{x_0^i})^{\frac{R_0f_i}{\beta }}, \end{aligned}$$

where *g* can be chosen by the adversary and $x_0$ is known. Similar is true for the signature element *B*. Signature elements *C* and *D* can be expressed as

$$\begin{aligned} C = g^{R_n[h(x)f_0 - f(x)h_0]} = B^{f'_0}\prod _{i=0}^{\lambda }(g^{x_0^i})^{\frac{R_nh_0}{\alpha }f_i'}, \end{aligned}$$

and

$$\begin{aligned} D= g^{R_0[h(x)f_{\lambda }-f(x)h_{\lambda }]} = A^{h'_\lambda }\prod _{i=0}^{\lambda }(g^{x_0^i})^{\frac{R_0f_\lambda }{\beta }h_i'}. \end{aligned}$$

Suppose that the adversary already obtained values $\frac{R_0f_i}{\beta }$, and $\frac{R_nh_i}{\alpha }$ $\forall i \in \{0, \dots , \lambda \},$ then to produce elements *C* and *D* with this information the adversary only needs the values $f'_i$ and $h'_i$ $\forall i \in \{0, \dots , \lambda \}.$

Alternatively, the attacker might want to obtain values

$$\begin{aligned} \alpha f_i = f'_i, \beta h_i = h'_i, \frac{R_0f'_i}{\alpha \beta } =R'_0f'_i, \frac{R_nh'_i}{\alpha \beta }=R'_nh'_i \end{aligned}$$

with $$R'_0 = \frac{R_0}{\alpha \beta }$$ and $$R'_n = \frac{R_n}{\alpha \beta }$$.

For $i \in \{0, \dots , \lambda \}$, an optimal way to obtain these values is to look for $f'_i, h'_i, R'_0, R'_n$ separately as they comprise $2(\lambda +1)+2$ elements. Alternatively, it is possible to combine values $\alpha \beta$ and look for $f'_i, h'_i, \alpha \beta , R_0, R_n$.

In the framework of MPPK/DS^6^, the public key components of MPPK/DS are even integers defined over the ring of integers $\mathbb {Z}/(p-1)\mathbb {Z} = \mathbb {Z}/2^{x}q\mathbb {Z}$, so the inverse elements of the public key components do not exist in the ring $\mathbb {Z}/2^{x}q\mathbb {Z}$. Hence, an adversary trying to perpetrate an attack on the public key needs to work in a different set such as $\mathbb {F}_q.$ The optimized version of MPPK/DS leverages the same mathematical property. Thus, the malicious party can not directly attack the public key in the framework of the optimized MPPK/DS. As an approach, the adversary can consider the public key elements modulo *q* since the ring $\mathbb {Z}/2^{x}q\mathbb {Z} \cong \mathbb {Z}/2^{x}\mathbb {Z} \times \mathbb {Z}/q\mathbb {Z},$ and then lift the results to the ring $\mathbb {Z}/2^{x}q\mathbb {Z}.$ Note that a single value modulo *q* is an entire equivalence class when considered modulo $2^{x}q$. Thus, the adversary needs to either verify that the lifted value is correct or the attack must be non-deterministic.

#### Proposition 3.1

*Let* $\lambda >1$ *and* $n \ge \lambda$. *Let* $\lambda +1 > m$. *There exists a probabilistic key recovery attack on MPPK/DS with classical non-deterministic computational complexity of* ${\mathscr {O}}([\sqrt{p}\log ^2p]q^{\lambda +2-m}2^{x[\lambda +3]}).$

#### *Proof*

Let $\lambda >1$ and $n \ge \lambda$. Let $\lambda +1 > m$. Consider a system of equations, formed by the coefficients of the public key polynomial ${\mathscr {P}}(x_0, x_1, \dots , x_m)$ over $\mathbb {F}_q$ for a fixed *j*. Using Gaussian elimination on variables $c'_{ij}=R_0c_{ij}$ from bottom to top, and leveraging the fact that values $R_0c_{0j}={\mathscr {N}}_{0j}$ are known as coefficients of the noise function, the system of equations can be reduced to a single equation of the form

$$\begin{aligned} F_{j}(f'_0, f'_1, \dots , f'_{\lambda }) = 0, \end{aligned}$$

for $f'_i = \alpha f_i$ with $i=0, 1, \dots , \lambda$. This process can be repeated for all other values of $j \in \{1, \dots , m\}.$ Thus, we can produce *m* equations in $\lambda +1$ variables.

This system of the equations of the form $F_{j}(f'_0, f'_1, \dots , f'_{\lambda })$ is underdetermined. We can reduce the system even further using Gaussian elimination to arrive at a single equation

$$\begin{aligned} {\mathscr {F}}(f'_0, f'_1, \dots , f'_{\lambda +1-m})=0. \end{aligned}$$

This is a typical Modular Diophantine Equation, and classical computational complexity of solving it is ${\mathscr {O}}(q^{\lambda +1-m})$ modulo *q*. The same Gaussian eliminations can be applied to the coefficients of the public key polynomial ${\mathscr {Q}}(x_0, x_1, \dots , x_m)$ over $\mathbb {F}_q$ for values *j* from 1 to *m*, with $h'_i=R_nh'_i$ $c''_{ij}=R_nc_{ij}$. We would have the following equation

$$\begin{aligned} {\mathscr {H}}(h'_0, h'_1, \dots , h'_{\lambda +1-m})=0. \end{aligned}$$

with the same complexity ${\mathscr {O}}(q^{\lambda +1-m})$ of solving it modulo *q*. Each solution set of $f'_0, \dots , f'_{\lambda }$ would give a solution set of $c'_{ij}$ for $i=1, 2, \dots , n$, and each solution set of $h'_0, \dots , h'_{\lambda }$ would give a solution set of $c''_{ij}$ for $i=1, 2, \dots , n.$ The relationship between $c'_{ij}$ and $c''_{ij}$ is $\frac{R_nc'_{ij}}{\alpha \beta } =\frac{R_0c''_{ij}}{\alpha \beta }$. Thus, the adversary looking for the value $R'_n = \frac{R_n}{\alpha \beta }$ can first find the value $R'_0 = \frac{R_0}{\alpha \beta }$ and use this relationship to discover $R'_n$ with classical complexity of ${\mathscr {O}}(\frac{1}{2}q)$ modulo *q*. The fraction comes form the fact that $R'_0$ and $R'_n$ are even numbers. Therefore, the total non-deterministic complexity for the solution set: $f'_0, \dots , f'_{\lambda }, h'_0, \dots , h'_{\lambda }, R_0, R_n$ is ${\mathscr {O}}(q^{\lambda +2-m})$ over $\mathbb {F}_q.$ By lifting all variables from $\bmod {q}$ to $\bmod {\varphi (p)}$, we would have a total non-deterministic complexity ${\mathscr {O}}(q^{\lambda +2-m}2^{x(\lambda +3)}).$

It should be understood that the attack would create a list of possible solution sets of $f'_0, \dots , f'_{\lambda }, h'_0, \dots , h'_{\lambda }, R'_0, R'_n$ with a list length $2q^{\lambda +2-m}2^{x(\lambda +3)}$. Of course, one of solution set from the list is the correct private key. The list can be shorten by utilizing intercepted signatures: $S_1=\{A_1, B_1, C_1, D_1\}, S_2=\{A_2, B_2, C_2, D_2\}, \dots , S_k=\{A_k, B_k, C_k, D_k\} \dots , S_K=\{A_K, B_K, C_K, D_K\}$. Using $A_k$ and $B_k$, one can create an equation

$$\begin{aligned} \bar{A}_kR_0h'(t_k) = \bar{B}_kR_nf'(t_k), \end{aligned}$$

with a purposely selected generator $\bar{g}\in \mathbb {F}_p,$ $\bar{A}_k=log_{\bar{g}}A_k$, $\bar{B}_k=log_{\bar{g}}B_k$ and $t_k$ for $x_0$ in the signature $S_k$. In a similar way, one can obtain another equation with $C_k$ and $D_k$

$$\begin{aligned} \bar{C}_kR_0[h'(t_k)f'_{\lambda }-f'(t_k)h'_{\lambda }] = \bar{D}_kR_n[h'(t_k)f'_0 - f'(t_k)h'_0]. \end{aligned}$$

Considered modulo *q*, the above two equations can be reduced to a single equation:

$$\begin{aligned} \bar{B}_k\bar{C}_kf'(t_k)[h'(t_k)f'_{\lambda }-f'(t_k)h'_{\lambda }] = \bar{A}_k\bar{D}_kh'(t_k)[h'(t_k)f'_0 - f'(t_k)h'_0] \bmod {q}. \end{aligned}$$

We did not find an efficient way to directly solve the above equations for $k=1, 2, \dots , K$, even for a large overdetermined equation system, except for the brute search. However, these equations obtained from the signatures could be used to verify all private key: $f'_0, \dots , f'_{\lambda }, h'_0, \dots , h'_{\lambda }$, obtained in the key recovery from the public key, and possibly produce a deterministic solution set of $f'_0, \dots , f'_{\lambda }, h'_0, \dots , h'_{\lambda }$, with a complexity ${\mathscr {O}}(\sqrt{p}\log ^2p)$, counting the complexity from the discrete logarithms. Then remaining unknowns $R'_0, R'_n$ make the attack still be probabilistic with overall complexity ${\mathscr {O}}([\sqrt{p}\log ^2p]q^{\lambda +2-m}2^{x[\lambda +3]}).$ The major contribution from using the signatures is the length of the possible solution sets being reduced from $2q^{\lambda +2-m}2^{x(\lambda +3)}$ to $q^{2}2^{2x+1}.$

In conclusion, combining signatures with the public key for the key recovery attack reduces a possible solution set, however, the computational complexity is higher. $\square$

#### Proposition 3.2

*Let* $\lambda >1$ *and* $n \ge \lambda$. *Let* $\lambda +1 > m$. *There exists a probabilistic key recovery attack on the Optimal MPPK/DS with classical complexity of* ${\mathscr {O}}(q^{\lambda +2-m}2^{x[\lambda +2]}).$ *Note that the difference between this attacking mechanism and the attack proposed in Proposition* 3.1*is that we do not make use of the relationship between the values* $R'_0$ *and* $R_n'$, *and do not use intercepted signatures to verify the solution. As a result the solution set produced with this attack is larger than that for attack* 3.1.

#### *Proof*

Let $\lambda >1$ and $n \ge \lambda$. Let $\lambda +1 > m$. Consider a system of equations, formed by the coefficients of the public key polynomial $P(x_0, x_1, \dots , x_m)$ over $\mathbb {F}_q$ for a fixed *j*. Using Gaussian elimination from bottom to top, and leveraging the fact that values $R_0c_{0j}$ are known as coefficients of the noise function, the system of equations can be reduced to a single equation of the form

$$\begin{aligned} F_{j}(f'_0, f'_1, \dots , f'_{\lambda }) = 0. \end{aligned}$$

Similarly for other *j* Thus, we can produce *m* equations in $\lambda +1$ variables. The values $f'_t$ are $\alpha f_t$ for all $t \in \{0, \dots , \lambda \}.$

Let $\lambda +1 > m$, then the system of the equations of the form $F_{j}(f_0, f_1, \dots , f_{\lambda })$ for all $j \ne 0$ is underdetermined. We can reduce the system even further using Gaussian elimination to arrive at a single equation

$$\begin{aligned} {\mathscr {F}}(f'_0, f'_1, \dots , f'_{\lambda +1-m})=0. \end{aligned}$$

This is a typical Modular Diophantine Equation, and classical computational complexity of solving it is ${\mathscr {O}}(q^{\lambda +1-m}).$ Now, the adversary needs to find $R'_0 = \frac{R_0}{\alpha \beta }$. This can be done using a brute force search over $\mathbb {F}_q$, with classical computational complexity of ${\mathscr {O}}(\frac{1}{2}q).$ All of the obtained values need to be lifted back to the ring $\mathbb {Z}/\varphi (p)\mathbb {Z}.$ The overall computational classical complexity to obtain $f'_t$ and $R'_0$ mod $\varphi (p)$ for all $t \in \{0, \dots , \lambda \}$ is ${\mathscr {O}}(\frac{1}{2}q^{\lambda +2-m}2^{x[\lambda +2]}).$

The exact same approach can be taken to obtain values $h'_{t}$ for $t \in \{0, \dots , \lambda \}$ and $R'_n = \frac{R_n}{\alpha \beta }$. Hence, the overall computational complexity of this probabilistic attack is ${\mathscr {O}}(q^{\lambda +2-m}2^{x[\lambda +2]}).$ $\square$

#### Proposition 3.3

*Let* $\lambda >1$, *and* $n \ge \lambda$. *Let* $\lambda + 1 > m$. *There exists a probabilistic key-recovery attack with classical complexity of* ${\mathscr {O}}(2\varphi (p)^{\lambda +2}2^{x})$.

#### *Proof*

Let $\lambda >1, n \ge \lambda$, and $\lambda +1 > m$. Coefficients of the public key polynomials $P(x_0, x_1, \dots , x_m)$ and $Q(x_0, x_1, \dots , x_m)$ for a fixed $j \ne 0$ form two systems of equations of the form $p_{kj} = \sum _{t+s = 1}^{n+\lambda -1}R_0\alpha f_{t}c_{sj}$ and $q_{kj} = \sum _{t+s = 1}^{n+\lambda -1}R_n \beta h_{t}c_{sj}$ over the ring $\mathbb {Z}/\varphi (p)\mathbb {Z}$. Using Gaussian elimination on $P(\cdot )$ from bottom to the top, this system of equations can be reduced to a single equation in $(\lambda +1)$ variables, namely $F_{j}(f'_0, f'_1, \dots , f'_{\lambda })=0,$ where $c'_{0j} = R_0c_{0j}$ is known as a coefficient of the noise function and $f'_{t} = \alpha f_{t}$ for all $t \in \{0, \dots , \lambda \}$. Similar can be applied to $Q(\cdot )$ with Gaussian elimination from top to bottom, yielding equation of the form $H_{j}(h'_0, h'_1, \dots , h'_{\lambda })=0,$ where $c'_{nj} = R_nc_{nj}$ is publicly known as a coefficient of the noise function and values $h'_{t} = \beta h_{t}$. These systems of equations are underdetermined. So the attacker needs to brute force search for values $f'_t$ and $h'_{t}$ for all $t \in \{1, \dots , \lambda \}$. These values then can be reduced together with the equations $F_{j}(f'_0, f'_1, \dots , f'_{\lambda })=0$ and $H_{j}(h'_0, h'_1, \dots , h'_{\lambda })=0$, and used to find $f'_0$ and $h'_0$ modulo *q*. Note that we can not solve for $f'_0, h'_0$ modulo $\varphi (p)$ since the coefficients of the function $F_{j}(f_0, f_1, \dots , f_{\lambda })=0$ are not co-prime with $\varphi (p).$ The classical complexity up to this step is ${\mathscr {O}}(2\varphi (p)^{\lambda }).$ In the field $\mathbb {F}_q$, the attacker can obtain values $f'_0, h'_0$. The adversary can then lift the values $f'_0, h'_0$ back to the ring modulo $\varphi (p).$ The complexity of this lift is ${\mathscr {O}}(2 \times 2^{x}).$ The adversary then needs to brute force search for the values $R'_0 = \frac{R_0}{\alpha \beta }, R'_n = \frac{R_n}{\alpha \beta }$ in the ring $\mathbb {Z}/\varphi (p)\mathbb {Z}$. Overall classical complexity is ${\mathscr {O}}(2\varphi (p)^{\lambda +2}2^{x})$. $\square$

#### Claim 3.4

*Let* $6+2\lambda +m > mn + 2m\lambda$, *then public key components considered individually or together form an underdetermined system of equations.*

#### *Proof*

The public key component ${\mathscr {P}}(x_0, x_1, \dots , x_m)$ forms a system of $m(n+\lambda -1)$ equations with $2+(\lambda +1)+m(n+1)$ variables to account for $\alpha , R_0, f_i \ \forall i \in \{0, \dots , \lambda \}$, and base polynomial coefficients $c_{lj}$ for $j \in \{1, \dots , m\}$ and $l \in \{0, \dots , n\}$. The same is true for the public key component ${\mathscr {Q}}(x_0, x_1, \dots , x_m).$ Noise functions ${\mathscr {N}}_0(x_1, \dots , x_m)$ and ${\mathscr {N}}_n(x_0, x_1, \dots , x_m)$ each forms a system of *m* equations in $m+1$ variables. Thus, considered individually each of the public key elements form an underdetermined system. Considered together, they form a system with $2m(n+\lambda -1)+2m$ equations and $2+2+2(\lambda +1)+m(n+1)$ variables to account for the common base polynomial coefficients. This system of equations is underdetermined when $2+2+2(\lambda +1)+m(n+1)>2m(n+\lambda -1)+2m$ or equivalently when $6+2\lambda +m>mn+2m\lambda .$ Otherwise, this system of equations is overdetermined and can be solved for the private key elements modulo *q*. $\square$

#### Proposition 3.5

*Let* $\lambda >1$ *and* $n \ge \lambda$. *Let* $\lambda +1 > m$. *There exists a probabilistic attack on the optimized MPPK/DS public key with classical complexity of* ${\mathscr {O}}(2q^{\lambda +2-m}2^{x(2(\lambda +1)+2)}).$

#### *Proof*

All the calculations in this attack are done modulo *q* except when explicitly specified otherwise. Note that the coefficients of the noise function of the form $R_0c_{0j} \forall j \in \{1, \dots , m\}$ are also components of the public key polynomial coefficients of ${\mathscr {P}}(\cdot )$, namely $p_{1j} = \alpha R_0c_{0j}f_1 + \alpha R_0c_{1j}f_0$. The adversary can take advantage of these shared components and reduce the number of variables. Indeed, the adversary can use Gaussian elimination until they arrive at the final equation with reduced number of variables. The final equation is homogeneous and will have variables $\alpha f_i = f'_i \ \forall i \in \{0, \dots , \lambda \}$ as well as $R_0c_{0j}$ which is known. This process can be repeated for other values *j* to reduce complexity of finding the values $\alpha f_i = f'_i \ \forall i \in \{0, \dots , \lambda \}$ to ${\mathscr {O}}(q^{\lambda +1-m}).$ Since, $n \ge \lambda$ and the value $R_0c_{0j}$ is known for all $j \in \{1, \dots , m\}$, the values $f'_i$ for all $i \in \{0, \dots , \lambda \}$ can be plugged in the equations generated by the public key polynomial coefficients of ${\mathscr {P}}(\cdot )$ to obtain values $R_0c_{ij}.$ Then a brute force search can be used to find $R_0' = \frac{R_0}{\alpha \beta }$ over the field $\mathbb {F}_q$ with classical complexity of ${\mathscr {O}}(\frac{1}{2}q).$ Similar attack on public key polynomial $Q(\cdot )$ can lead values $h'_i$ and $R'_n = \frac{R_n}{\alpha \beta }$. All of these values need to be lifted to the ring $\mathbb {Z}/\varphi (p)\mathbb {Z}$. Some of the obtained values can be verified using the following verification equation

$$\begin{aligned} R'_0f'(x_0){\mathscr {Q}} = R'_nh'(x_0){\mathscr {P}}, \end{aligned}$$

where we assume that the noise is set to zero. The lift has classical complexity of ${\mathscr {O}}(2^{x(2(\lambda +1)+2)}).$ This would potentially reduce the number of possible solutions significantly. The overall classical complexity of this attack is ${\mathscr {O}}(2q^{\lambda +2-m}2^{x(2(\lambda +1)+2)}).$ $\square$

#### Proposition 3.6

*There exists a non-deterministic attack on MPPK/DS with classical computational bit complexity of* ${\mathscr {O}}(\varphi (p)^{2(n+1)+1}(\log ^2p + \log p)2^{x(\lambda +1)+1}).$

#### *Proof*

The attack goes as follows. Fix $j=j_1$ and $j= j_2$. Consider coefficients of a public polynomial $P(x_0, x_1, \dots , x_m)$ of the form $\sum _{k=s+t}R_0\alpha f_{t}c_{sj}$ for all $t \in \{0, \dots , \lambda \}$ and $s \in \{0, \dots , n\}$. We will denote $\alpha f_{t} = f'_t$ and $\beta h_{t} = h'_{t}$ for all $t \in \{0, \dots , \lambda \}.$ The adversary can brute force search for values $c_{sj_1},$ and $c_{sj_2}$ for all $s \in \{0, \dots , n\}$ in the ring $\mathbb {Z}/\varphi (p)\mathbb {Z}.$ Classical computational bit complexity of this step is ${\mathscr {O}}(\varphi (p)^{2(n+1)}).$ These values are then used to solve for the coefficients of $R_0\alpha f(x_0)$ as

21 $$\begin{aligned} \begin{bmatrix} R_0f'_0\\ R_0f'_1\\ \vdots \\ R_0f'_{\lambda } \end{bmatrix} = \begin{bmatrix} c_{1j} &{} c_{0j} &{} \dots &{}0 &{}0\\ c_{2j} &{} c_{1j} &{} \dots &{} 0 &{} 0\\ \vdots &{} \vdots &{} \dots &{} \vdots &{} \vdots \\ c_{nj} &{} c_{(n-1)j} &{} \dots &{} c_{0j} &{} 0\\ 0 &{} c_{nj} &{} \dots &{} c_{1j} &{} c_{0j} \\ 0 &{} 0 &{} \dots &{} c_{2j} &{} c_{1j} \end{bmatrix}^{-1}\begin{bmatrix} p_{1j}\\ p_{2j}\\ p_{3j}\\ \vdots \\ p_{(n+\lambda -2)j}\\ p_{(n+\lambda -1)j} \end{bmatrix} \end{aligned}$$

for both $j = j_1$ and $j = j_2$. The same strategy is applied to the coefficients of the polynomial $Q(x_0, x_1, \dots , x_m)$ for $j = j_1$ and $j = j_2$. Computational complexity of these step is $(\log ^2 p + \log p)$. To verify the correct solution, the versifier can search for values $c_{sj}$ that yield the same $R_0f_{t}$ and $R_nh_{t}$ for $j=j_1$ and $j=j_2$. Note that generally, there might be more than a single solution that satisfies this property. However, we assume that the adversary is at the advantage and they are in the scenario where only a single such solution exists. In this case, the adversary determined values $R_0f'_{t}, R_nh'_{t}$ for $t \in \{0, \dots , \lambda \}$ and $c_{sj}$ for $s \in \{0, \dots , n\}$, $j = j_1$ and $j=j_2$. The adversary can leverage base polynomial coefficients considered in conjunction with noise functions to obtain $R_0$ as $\frac{n_{0j_1}}{c_{0j_1}}$ and $R_n$ as $\frac{n_{nj_1}}{c_{nj_1}}$. There are a few more pieces of information that the adversary needs, namely values $f'_t$ and $h'_t$ and the value $\alpha \beta .$ The adversary can reduce values $R_0f'_{t}$ and $R_nh'_{t}$ as well as $R_0, R_n$ mod *q* to calculate $f'_t$ and $h'_t$. These values then need to be lifted back to the ring $\mathbb {Z}/\varphi (p)\mathbb {Z}.$ Classical computational bit complexity of this step is ${\mathscr {O}}(2 \times 2^{x(\lambda +1)})$. The adversary can verify if the lift is successfully by comparing lifted values multiplied by $R_0$ and $R_n$ correspondingly to known values $R_0f'_{t}, R_nh'_{t}.$ Having values $f'_{t}, h'_{t}, R_0, R_n$, the adversary need the value $\alpha \beta$ which they can find using brute force search over the ring $\mathbb {Z}/\varphi (p)\mathbb {Z}.$ The overall complexity of this attack is ${\mathscr {O}}(\varphi (p)^{2(n+1)+1}(\log ^2p + \log p)2^{x(\lambda +1)+1}).$ $\square$

#### Proposition 3.7

*There exists a non-deterministic attack on MPPK/DS with classical complexity of* ${\mathscr {O}}(2q^{4-(\lambda +1)}2^{x(\lambda +1)}\varphi (p))$, *when* $n=2$ *and* $\lambda < 3$.

#### *Proof*

The number of public key equations produced using coefficients of the polynomial ${\mathscr {P}}(x_0, x_1, \dots , x_m)$ is $n+\lambda -1$ for a given *j*. When we choose $n=2$, the number of public key equations becomes $\lambda +1$, which is equal to the number of coefficients of the private univariate polynomial $f(x_0)$ or $h(x_0)$. Under this consideration, we can establish the following equation with public key coefficients for $j=j_1$ and $j=j_2$

22 $$\begin{aligned} \begin{bmatrix} c'_{1j_1} &{} c'_{0j_1} &{} \dots &{}0 &{}0 &{}0\\ c'_{2j_1} &{} c'_{1j_1} &{} \dots &{} 0 &{}0 &{} 0\\ \vdots &{} \vdots &{} \dots &{} \vdots &{} \vdots &{} \vdots \\ 0 &{} 0 &{} \dots &{} c'_{2j_1} &{}c'_{1j_11} &{} c'_{0j_1} \\ 0 &{} 0 &{} \dots &{} 0 &{} c'_{2j_1} &{} c'_{1j_1} \end{bmatrix}^{-1}\begin{bmatrix} p_{1j_1}\\ p_{2j_1}\\ \vdots \\ p_{\lambda j_1}\\ p_{(\lambda +1)j_1} \end{bmatrix} = \begin{bmatrix} c'_{1j_2} &{} c'_{0j_2} &{} \dots &{}0 &{}0 &{}0\\ c'_{2j_2} &{} c'_{1j_2} &{} \dots &{} 0 &{}0 &{} 0\\ \vdots &{} \vdots &{} \dots &{} \vdots &{} \vdots &{} \vdots \\ 0 &{} 0 &{} \dots &{} c'_{2j_2} &{}c'_{1j_2} &{} c'_{0j_2} \\ 0 &{} 0 &{} \dots &{} 0 &{} c'_{2j_2} &{} c'_{1j_2} \end{bmatrix}^{-1}\begin{bmatrix} p_{1j_2}\\ p_{2j_2}\\ \vdots \\ p_{\lambda j_2}\\ p_{(\lambda +1)j_2} \end{bmatrix}, \end{aligned}$$

where $c'_{ij} = R_0c_{ij}.$ The above matrix equation can be expanded into $\lambda + 1$ equations with 4 unknowns: $c'_{1j_1}, c'_{2j_1}, c'_{1j_2}, c'_{2j_2},$ with $c_{01} = R_0{\mathscr {N}}_{01}$ and $c'_{02} = R_0{\mathscr {N}}_{02}$. Let $\lambda < 3$. Due to the fact of all public key coefficients are even integers, we can only carry out the solution for $\bmod {q}$ with a complexity ${\mathscr {O}}(q^{4-(\lambda +1)})$. With the knowledge of the values $c'_{11}, c'_{21}, c'_{12}, c'_{22}$, we can then solve for $f'_0, f'_1, \dots , f'_{\lambda }$ modulo *q*, where $f'_{t} = \alpha f_t$ for all $t \in \{0, \dots , \lambda \}$ as follows

23 $$\begin{aligned} \begin{bmatrix} f'_{0}\\ f'_{1}\\ \vdots \\ f'_{\lambda -1}\\ f'_{\lambda } \end{bmatrix}=\begin{bmatrix} c'_{1j_1} &{} c'_{0j_1} &{} \dots &{}0 &{}0 &{}0\\ c'_{2j_1} &{} c'_{1j_1} &{} \dots &{} 0 &{}0 &{} 0\\ \vdots &{} \vdots &{} \dots &{} \vdots &{} \vdots &{} \vdots \\ 0 &{} 0 &{} \dots &{} c'_{2j_1} &{}c'_{1j_1} &{} c'_{0j_1} \\ 0 &{} 0 &{} \dots &{} 0 &{} c'_{2j_1} &{} c'_{1j_1} \end{bmatrix}^{-1}\begin{bmatrix} p_{1j_1}\\ p_{2j_1}\\ \vdots \\ p_{\lambda j_1}\\ p_{(\lambda +1)j_1} \end{bmatrix}. \end{aligned}$$

The obtained solution can be verified using similar equation system for $j = j_2.$ Note that these solutions were obtained modulo *q*, and need to be lifted to the ring $\mathbb {Z}/\varphi (p)\mathbb {Z}.$ The complexity of the lifting step is ${\mathscr {O}}(2^{x(\lambda +1)}).$ The adversary still needs the value $R_0' = \frac{R_0}{\alpha \beta }$ which can be found using brute force search over $\mathbb {Z}/\varphi (p)\mathbb {Z}.$ All the steps can be repeated to find values $h'_{t} = \beta h_t$ for all $t \in \{0, \dots , \lambda \}$ and $R'_n.$ The overall complexity of this attack is ${\mathscr {O}}(2q^{4-(\lambda +1)}2^{x(\lambda +1)}\varphi (p))$. $\square$

Many of the attacks that we have discovered on the original MPPK/DS scheme^6^, also apply to the optimized version of the MPPK signature scheme. For reasons of simplicity, we will direct the reader to a detailed description of the given attacks in^6^ and give their classical complexities when considered in the framework of the optimized MPPK/DS.

#### Proposition 3.8

*There exists an attack on the public key of the MPPK/DS optimized signature scheme with classical complexity of* ${\mathscr {O}}([q^{(\lambda +2)}+q]2^{x(2(\lambda +1)+2)}).$

#### *Proof*

The attack is described in detail in Claim 4.7 of MPPK/DS^6^. All of the arithmetic is done modulo *q* unless stated otherwise. To adapt this attack to the optimized version we point out that after a brute force search for $R_0$ the matrices considered are

$$\begin{aligned} \begin{bmatrix} \alpha f_1 &{} \alpha f_0 &{} 0 &{} \dots &{} 0\\ \alpha f_2 &{} \alpha f_1 &{} \alpha f_0 &{} \dots &{} 0\\ \vdots &{} \vdots &{} \vdots &{} \vdots &{} \vdots \\ 0 &{}\alpha f_{\lambda }&{} \alpha f_{\lambda -1} &{} \dots &{} \alpha f_0 \\ 0 &{} 0 &{} \alpha f_{\lambda } &{} \dots &{} \alpha f_1 \end{bmatrix} \begin{bmatrix} c_{0j}\\ c_{1j}\\ \vdots \\ c_{nj}\\ 0 \\ \vdots \\ 0 \end{bmatrix} = \begin{bmatrix} p'_{1j}\\ p'_{2j}\\ p'_{3j}\\ \vdots \\ p'_{(n+\lambda -1)j} \end{bmatrix}. \end{aligned}$$

with $p'_{kj} = \frac{1}{R_0}p_{kj}$. The adversary then uses brute force search to find values $\alpha f_{i} \ \forall i \in \{0, \dots , \lambda \}.$ The complexity at this stage is ${\mathscr {O}}(q^{1+(\lambda +1)}).$ Once the base polynomial coefficients are found and used to find the values $\beta R_{n} h_{i} \ \forall i \in \{0, \dots , \lambda \}$, the adversary can also use the base polynomial coefficients together with the noise functions to find values $R_{0}$ and $R_{n}$. As stated in^6^, Claim 4.7, all of this values are found modulo *q*. The adversary then needs to find the value $\alpha \beta .$ To do that, the adversary can brute force search for the value $\alpha \beta .$ All of the solutions need to be lifted to the ring $\mathbb {Z}/(p-1)\mathbb {Z}$, the adversary can follow the same steps as described in Proposition 3.5. In fact, instead of lifting $R_0, R_n, \alpha \beta$ separately, the attacker can lift $R_0' = \frac{R_{0}}{\alpha \beta }, R'_{n}= \frac{R_{n}}{\alpha \beta }$. The overall complexity then is ${\mathscr {O}}([q^{(\lambda +2)}+q]2^{x(2(\lambda +1)+2)}).$ $\square$

#### Proposition 3.9

*Let* $n <5$. *There exists an attack on the noise functions of the optimized MPPK/DS with classical complexity of* ${\mathscr {O}}(q^{4}2^{x(2(\lambda +1)+3)}).$

#### *Proof*

As we have mentioned in Claim 3.4, noise functions ${\mathscr {N}}_0(\cdot )$ and ${\mathscr {N}}_n(\cdot )$ each form systems of *m* equations in $m+1$ variables. Considered together, they form a system of 2*m* equations in $2m+2$ variables. This system is underdetermined. Suppose that the adversary considers this system modulo *q*. They can brute force search for 2 values in the field $\mathbb {F}_q$, say $R_{0}$ and $R_{n}$. The system then transforms to one with 2*m* equations in 2*m* variables, and can be solved for the base polynomial coefficients $c_{0j}$ and $c_{nj}$ for all $j \in \{1, \dots , m\}.$ The base polynomial coefficients as well as values $R_0$ and $R_n$ can then be used to solve for the unknowns $f'_i, h'_i$ using system of equations generated by ${\mathscr {P}}(\cdot )$ and ${\mathscr {Q}}(\cdot ).$ Note that the system of equations generated by ${\mathscr {P}}$ and/or ${\mathscr {Q}}$ even when values $R_0, R_n, c_{0j}, c_{nj}$ are substituted remains underdetermined. Thus, a brute force search is needed with complexity ${\mathscr {O}}(q)$. The adversary then needs to find values $\alpha \beta$ using brute force search. All of the obtained values are in the field $\mathbb {F}_q$ and need to be lifted to the ring $\mathbb {Z}/\varphi (p)\mathbb {Z}$. The lifting can be done using the same technique as described in the proof of Proposition 3.5. The total classical complexity of this attack is then ${\mathscr {O}}(q^{4}2^{x(2(\lambda +1)+3)}).$ $\square$

#### Proposition 3.10

*Let* $\lambda >1$. *Let* $\lambda +2 > m$. *There exists an attack on the the optimized MPPK/DS with classical complexity of* ${\mathscr {O}}(q^{\lambda +2-m}2^{x(2(\lambda +1)+2)}\varphi (p)).$

#### *Proof*

Let $\lambda >1$. Let $\lambda +2 > m$. Begin the attack by considering the public coefficients of the public key polynomial ${\mathscr {P}}(x_0, \dots , x_m)$ for a fixed value *j*. We can use Gaussian elimination on them from bottom to top to eliminate base polynomial coefficients $c'_{ij} = R_0c_{ij}.$ Moreover, we can use the fact that $R_0c_{ij}$ is a public coefficient of the noise function ${\mathscr {N}}.$ This would produce a single equation of the form

$$\begin{aligned} F_{j}(f'_0, f'_1, \dots , f'_{\lambda }), \end{aligned}$$

where the values $f'_{t} = \alpha f_{t}.$ This process can be repeated for other values of *j*. This would generate *m* equations of the form $F_{j}(f'_0, f'_1, \dots , f'_{\lambda }, \alpha )$. Gaussian elimination can be used again to reduce the system of such equations to a single equation

$$\begin{aligned} {\mathscr {F}}_{j}(f'_0, f'_1, \dots , f'_{\lambda +1-m}). \end{aligned}$$

We can solve this equation with classical complexity of ${\mathscr {O}}(q^{\lambda +1-m}).$ The produced values can then be used to find values $R_0c_{ij}.$ We then brute force search for the value $R_0$. The base polynomial coefficients can then in turn used to find $R_n$ from the coefficients of the noise function. Moreover, they can be used to find values $\beta R_n h_{t}$ for all $t \in \{0, \dots , \lambda \}.$ All of these values need to be lifted. The complexity of the lift is ${\mathscr {O}}(2^{x(2(\lambda +1)+2)})$. We can use the technique outlined in Proposition 3.5 to verify these solutions. However, first we need to brute force search for the value $\alpha \beta$. The overall classical complexity is ${\mathscr {O}}(q^{\lambda +2-m})2^{x(2(\lambda +1)+2)}\varphi (p)).$ $\square$

We conclude, that the best attacking mechanisms that we have discovered to this day on the public key are described in Proposition 3.1, and Proposition 3.2 with classical complexity of ${\mathscr {O}}([\sqrt{p}\log ^2p]q^{\lambda +2-m}2^{x[\lambda +3]})$ and ${\mathscr {O}}(q^{\lambda +2-m}2^{x[\lambda +2]})$ respectively.

### Private key recovery from the signature

The attacks given in this section have been first introduced in the Section “Security of the private key given the signature.”^6^. We adapted these attacks to the optimized version of MPPK/DS. Thus, we will not describe them in detail but rather refer the reader to the attack described in detail in^6^ and state new complexity corresponding to the Optimized MPPK/DS.

#### Proposition 3.11

*There exists an attack on the optimized version of MPPK/DS using signatures, obtained from communication records, and public key. Classical complexity of this attack is* ${\mathscr {O}}(2(2\lambda +1)(\sqrt{p}\log p)q^{2}2^{x(2(\lambda +1)+2)}\varphi (p)).$

#### *Proof*

The attack is described in detail in^6^, Proposition 4.12. The initial step of this attack remains the same in the framework of the optimized MPPK/DS, however, the values $f'_{t}$ and $h'_{t}$ are not $R_0f_t$ and $R_nh_t$ but rather $f'_t = \alpha R_0 f_t$ and $h'_t = \beta R_n h_t$ for all $t \in \{0, \dots , \lambda \}$. Classical complexity to find the values $f'_t$ and $h'_t$ remains $2(2\lambda +1)q\sqrt{p}\log p$ for the optimized MPPK/DS. These values are used to find base polynomial coefficients, as shown in^6^, Proposition 4.12, which in turn are used together with noise coefficients to find $R_0$ and $R_n$ modulo *q*. So far, the attack was carried out modulo *q*, and the adversary needs to lift the private key values to the ring $\mathbb {Z}/\varphi (p)\mathbb {Z}.$ The attacker then needs to find $\alpha \beta$ using brute force search. The total classical complexity of this attack is then ${\mathscr {O}}(2(2\lambda +1)q\sqrt{p}\log p 2^{x(2(\lambda +1)+2)}\varphi (p)).$ $\square$

#### Proposition 3.12

*There exists an attack on the optimized version of MPPK/DS using signatures obtained from communication records. Classical complexity of this attack is* ${\mathscr {O}}(4(\lambda +1)p^{\lambda +1}2^{x(4\lambda +4)}\sqrt{p}\log p).$

#### *Proof*

The attack is described in detail in^6^, Proposition 4.13. To adapt this attack to the Optimized MPPK/DS, note that $f'_t = \frac{R_0f'_t}{\beta }$ and $h'_t = \frac{R_nh'_t}{\alpha }$ in this case. The complexity of finding all the values needed to create signature components *A*, *B*, *C*,  and *D* modulo *q* remains $4(\lambda +1)p^{(\lambda +1)}\sqrt{p}\log p$. The lifting step has different complexity for the optimized DS. We lift the values altogether. The classical complexity of this step is $2^{x(4\lambda +4)}$. The total classical complexity is then ${\mathscr {O}}(4(\lambda +1)p^{\lambda +1}\sqrt{p}\log p2^{x(4\lambda +4)}).$ $\square$

Note that it is possible to combine key-recovery attacks and attacks that use intercepted signatures. The best such attack is given as Proposition 3.1. We showed, however, that to the best of our knowledge, this combination does not benefit the attacker. In particular, we believe that using intercepted signatures reduces the number of possible solutions, however it increases the complexity of the attack. In part, this is due to a fact that each signature is associated with a new base *g*, which introduces a new unknown variable for every signature considered. To eliminate *g*, the attacker has to combine signature elements. This combination increases the complexity during the lifting process.

We conclude that the most optimal attack on the signature in the framework of the Optimized MPPK/DS has classical complexity of ${\mathscr {O}}(2(2\lambda +1)q\sqrt{p}\log p 2^{x(2(\lambda +1)+2)}\varphi (p))$ as described in Proposition 3.11.

### Spoofing attacks

Here we describe the most optimal direct spoofing attack that we have discovered on the Optimized MPPK/DS. This attack is inspired by the attacking mechanism described in^6^, Proposition 4.15. Note that this attack does not apply to the original version of the MPPK/DS signature scheme, as described in^6^, due to the signature element *E*.

#### Proposition 3.13

*The best direct spoofing attack on the Optimized MPPK/DS scheme has classical complexity of* ${\mathscr {O}}(m p^{4})$ *in terms of modular exponentiation over* $\mathbb {F}_p$ *or* ${\mathscr {O}}(m (log_2 p)^{12} p^{4})$ *in terms of bit operations*.

#### *Proof*

The attack is inspired by^6^, Proposition 4.15. The adversary must guarantee that malicious signature values *A*, *B*, *C*, *D* satisfy $A^{\bar{{\mathscr {Q}}(\cdot )}} = B^{\bar{{\mathscr {P}}(\cdot )}}C^{\bar{{\mathscr {N}}_0(\cdot )}}D^{\bar{{\mathscr {N}}_n(\cdot )}}.$ Here, the public key polynomials can be expanded as

$$\begin{aligned}{} & {} \bar{{\mathscr {P}}}(x_0, x_1, \dots , x_m) = \sum _{i=1}^{m}{\mathscr {P}}_{i}x_i, \\{} & {} \quad \bar{{\mathscr {Q}}}(x_0, x_1, \dots , x_m) = \sum _{i=1}^{m}{\mathscr {Q}}_{i}x_i, \\{} & {} \quad \bar{{\mathscr {N}}_0}(x_0, x_1, \dots , x_m) = \sum _{i=1}^{m}{\mathscr {N}}_{0i}x_i, \\{} & {} \quad \bar{{\mathscr {N}}_n}(x_0, x_1, \dots , x_m) = \sum _{i=1}^{m}{\mathscr {N}}_{ni}x_i, \end{aligned}$$

where ${\mathscr {P}}_{i}, {\mathscr {Q}}_{i}, {\mathscr {N}}_{0i}, {\mathscr {N}}_{ni}$ are functions of $x_0$. The verification expression then becomes

$$\begin{aligned} A^{\sum _{i}^{m}{\mathscr {Q}}_ix_i} = B^{\sum _{i}^{m}{\mathscr {P}}_ix_i}C^{\sum _{i}^{m}{\mathscr {N}}_{0i}x_i}D^{\sum _{i}^{m}{\mathscr {N}}_{ni}x_i}, \end{aligned}$$

for all $i \in \{1, \dots , m\}.$ It is true that if $A^{{\mathscr {Q}}_i} = B^{{\mathscr {P}}_i}C^{{\mathscr {N}}_{0i}}D^{{\mathscr {N}}_{ni}}$ for every $i \in \{1, \dots , m\}$, then $(A^{{\mathscr {Q}}_i})^{x_{i}} = (B^{{\mathscr {P}}_i}C^{{\mathscr {N}}_{0i}}D^{{\mathscr {N}}_{ni}})^{x_i}$ for every $i \in \{1, \dots , m\}$. It is also true that if $(A^{{\mathscr {Q}}_i})^{x_{i}} = (B^{{\mathscr {P}}_i}C^{{\mathscr {N}}_{0i}}D^{{\mathscr {N}}_{ni}})^{x_i}$, then $\prod _{i=1}^{m}(A^{{\mathscr {Q}}_i})^{x_{i}} = \prod _{i=1}^{m}(B^{{\mathscr {P}}_i}C^{{\mathscr {N}}_{0i}}D^{{\mathscr {N}}_{ni}})^{x_i},$ which can be re-written as

$$\begin{aligned} A^{\sum _{i}^{m}{\mathscr {Q}}_ix_i} = B^{\sum _{i}^{m}{\mathscr {P}}_ix_i}C^{\sum _{i}^{m}{\mathscr {N}}_{0i}x_i}D^{\sum _{i}^{m}{\mathscr {N}}_{ni}x_i}, \end{aligned}$$

for all $i \in \{1, \dots , m\}.$ Thus, if the adversary can find values *A*, *B*, *C*, *D* such that $A^{{\mathscr {Q}}_i} = B^{{\mathscr {P}}_i}C^{{\mathscr {N}}_{0i}}D^{{\mathscr {N}}_{ni}}$ for every $i \in \{1, \dots , m\}$, then these values can be used to spoof the signature regardless of the choice of noise variables $x_1, \dots , x_m.$ The classical complexity of this attack is ${\mathscr {O}}(m \times p^{3+\delta })$, where ${\mathscr {O}}(m \times p^3)$ comes from brute force search for values *B*, *C*, *D*, and *A* can be calculated as $A^{Q_i} = {\mathscr {C}}$, where ${\mathscr {C}}$ is the constant produced by $B^{{\mathscr {P}}_i}C^{{\mathscr {N}}_{0i}}D^{{\mathscr {N}}_{ni}}.$ The calculation of *A* we estimate to increase the complexity to ${\mathscr {O}}(p^{3+\delta })$. Here, $\delta = 1$ if the technique to obtain *A* is brute-force search using classical computers. In this case, the classical complexity is ${\mathscr {O}}(mp^{4})$ with classical computers. The complexity of this attack on a quantum computer can be significantly reduced due to Shor’s algorithm with $\delta =0$. We then obtain the complexity to be ${\mathscr {O}}(\sqrt{mp^3})$.

In NIST PQC security description^39^, it should be noted that NIST is primarily concerned with attacks that use classical (rather than quantum) queries to the signing oracle. We interpret it as NIST being primarily concerned with the security of private keys rather than the spoofing attacks because spoofing must be performed per signing message which is not efficient. Based on this consideration, we set the complexity of MPPK/DS to ${\mathscr {O}}(mp^4)$ operations of modular exponentiation.

The time complexity of spoofing can be calculated based on the complexity of the bit operation of the modular exponentiation: ${\mathscr {O}}((log_2p)^3)$. We have total $m\times 4$ modular exponentiation evaluations so the overall time complexity is ${\mathscr {O}}(m \ (log_2 \ p)^{12} p^4)$ $\square$

### Security conclusion

We have discovered four different ways to attack the Optimized MPPK/DS scheme, namely key-recovery attack using the knowledge of a public key, key-recovery attack using the knowledge of the signature, a combination of the two, and direct spoofing. In most cases, the adversary can not solve for any private information modulo $\varphi (p)$ directly due to even coefficients of the public key elements, thus the attacker is reduced to solving for the private key elements modulo *q*, and then lifting the solutions back to the ring $\mathbb {Z}/\varphi (p)\mathbb {Z}.$ Hence, in most cases the best complexity of the attack has form ${\mathscr {O}}(q^{r}2^{xs}),$ where the values *r*, *s* depend on the security parameters $n,m,\lambda .$ Otherwise, the adversary can choose to brute force for some private key values but that would also lead to a high complexity of the form ${\mathscr {O}}(\varphi (p)^{r}),$ where $r = poly(n,m,\lambda ).$ Note also that in most cases the adversary is faced with an underdetermined system of equations, and thus, is required to use brute force search for some values of the private key.

We provide the reader with Table 1 summarizing classical complexity of the best attacks we have discovered on the Optimized MPPK/DS scheme up to this date.

**Table 1:** Classical complexity of the best attacks we have discovered on the Optimized MPPK/DS scheme to this date.

Proposition #	Classical complexity
3.1	${\mathscr {O}}([\sqrt{p}\log ^2p]q^{\lambda +2-m}2^{x[\lambda +3]})$
3.2	${\mathscr {O}}(q^{\lambda +2-m}2^{x[\lambda +2]})$
3.11	${\mathscr {O}}(2(2\lambda +1)q\sqrt{p}\log p 2^{x(2(\lambda +1)+2)}\varphi (p))$
3.13	${\mathscr {O}}(m \ (log_2 \ p)^{12} p^4)$

Note that Optimized MPPK/DS as well as the original MPPK/DS schemes are resistant to the known quantum attacks such as attacks using Shor’s algorithm. However, the attacker can benefit from using Shor’s or Grover’s algorithm to gain a better attacking complexity. For most of the attacks we have discovered, Grover’s algorithm can be used to improve the brute force search component of the attacks. That would bring a square root speed up to the attack. We provide the reader with Table 2 illustrating quantum complexity of the best attacks we have discovered on the Optimized MPPK/DS.

**Table 2:** Quantum complexity of the best attacks we have discovered on the Optimized MPPK/DS scheme to this date.

Proposition #	Classical complexity
3.1	${\mathscr {O}}(\sqrt{q^{\lambda +2-m}2^{x[\lambda +3]}})$
3.2	${\mathscr {O}}(\sqrt{q^{\lambda +2-m}2^{x[\lambda +2]}})$
3.11	${\mathscr {O}}(\sqrt{q2^{x(2(\lambda +1)+2)}\varphi (p))}$
3.13	${\mathscr {O}}(m \ (log_2 \ p)^{12} \sqrt{p^4})$

**Author note** We have recently became aware of an algebraic attack on MPPK/DS proposed by Hao Guo^40^. The authors acknowledge this attack and are currently making modifications to the MPPK/DS algorithm to withstand this proposed attack. It's important to note that our aim is to maintain the main structure of MPPK/DS, while also securing it against the proposed algebraic attack and other similar attacks.

## Discussions

### Sizes of public key, private key, and signature

As we have shown in “Security conclusion”, the best key-recovery attacks on the public key in the framework of the Optimized MPPK/DS has classical complexity of ${\mathscr {O}}([\sqrt{p}\log ^2p]q^{\lambda +2-m}2^{x[\lambda +3]})$ and ${\mathscr {O}}(q^{\lambda +2-m}2^{x[\lambda +2]})$ as shown in Propositions 3.1 and  3.2 respectively. The best attack we have discovered on the signature has classical complexity of ${\mathscr {O}}(2(2\lambda +1)q\sqrt{p}\log p 2^{x(2(\lambda +1)+2)}\varphi (p))$ as described in Proposition 3.11. The best direct spoofing attack has classical complexity of ${\mathscr {O}}(m \ (log_2 \ p)^{12}p^{4})$ as described in Proposition 3.13.

Although two extra private values $\alpha$ and $\beta$ are introduced, the private key would not be increased in its size because the private key elements can be replaced with $f'_i = \alpha f_i, h'_i=\beta h'_i, R'_0= \frac{R_0}{\alpha \beta }, R'_n= \frac{R_n}{\alpha \beta }$. The size of the public key can be calculated as $2m(n+\lambda )$ field elements. The signature size can be calculated as $4 \times M$, where *M* is the number of message segments to be signed. Based on the most efficient discovered optimal attacks given in Table 1, we provide Table 3 illustrating sample parameters of the Optimized MPPK/DS scheme configured to provide NIST security levels I, III, and V while preventing any of the discovered attacks. Indeed, the classical complexity of the remaining attacks that we have discovered is larger. We offer two categories of configurations: maximum secure or Xsecure with 384 bits of entropy for all levels, and constrained secure for IoT devices with 192 bits of entropy for level I and III and 256 bits of entropy for level V. To allow for randomized verification, the number of noise variables is set to $m = 2$.

**Table 3:** We propose configuration of the Optimized MPPK/DS, and provide key and signature sizes as well as entropy based on the best discovered attack that correspond to the proposed parameter sets.

${\mathscr {O}}(q^{\lambda +2-m}2^{x[\lambda +2]})$ ${\mathscr {O}}([\sqrt{p}\log ^2p]q^{\lambda +2-m}2^{x[\lambda +3]})$		Level I	Level III	Level V
Xsecure	(log q).x.n.$\lambda$.m	64.64.2.2.2	64.64.2.2.2	64.64.2.2.2
Private key (B)		128	128	128
Public key (B)		256	256	256
Signature (B)	SHA-256	128	128	128
Signature* (B)	SHA-256, -384, -512	128	192	256
Equivalent entropy (bits)$^{*}$		384	384	384
Constrained	(log q).x.n.$\lambda$.m	32.32.2.2.2	32.32.2.2.2	32.32.3.3.2
Private key (B)		64	64	80
Public key (B)		128	128	192
Signature (B)	SHA-256	128	128	128
Signature* (B)	SHA-256, -384, -512	128	192	256
Equivalent entropy (bits)$^{*}$		192	192	256

$^{*}$Based on the best complexity among the two calculated.

Public key sizes, private key sizes, and signature sizes of the Optimized MPPK/DS are characterized by the security parameters $\log p, \log q, n, \lambda , m$ for NIST levels I, III, and V corresponding to all the discovered attacks.

The MPPK-Xsecure category is selected to be a single configuration $(log q).x.n.\lambda .m=64.64.2.2.2$ for all three NIST security levels, choosing the prime field of 128 bits with a sub-prime *q* to be 64 bits, quadratic polynomials with respect to the message variable $x_0$, two noise variables $x_1, x_2$. Based on the optimal attack in Proposition 3.2, it offers 384 bits of entropy with a public key being 256 bytes, private key being 128 bytes, and signature at 128 bytes if SHA-256 is used or 192 bytes if SHA-384 is used, or 256 bytes if SHA-512 is used. For the resource constrained devices, the MPPK-Constrained category uses the prime field of 64 bits with a sub-prime *q* being 32 bits. The configuration 32.32.2.2.2 works for NIST security level I and III holding a 192 bits of entropy with the public key size being 128 bytes, private key size being only 64 bytes, and signature size being 128 bytes for SHA-256, or 192 bytes for SHA-384. We choose a configuration 32.32.3.3.2 or cubic polynomials for the message variable for NIST security V offering 256 bits of entropy and 80-byte private key, 192-byte public key, 128- (SHA256) or 256- (SHA512) byte signature.

### Comparisons

In this subsection, we compare Optimized MPPK/DS with NIST standardized algorithms: Dilithium, Falcom, and SPHINCS+ for key sizes and signature sizes. Table 4 illustrates the size comparisons with the original MPPK/DS^6^ and NIST standardized algorithms.

We state that the security analysis published in our original paper MPPKS/DS^6^ lacks newly discovered attacks. Since the publication of MPPK/DS we have discovered some new attacking mechanisms. In particular, attacking mechanisms outlined in Proposition 3.2 and Proposition 3.1 apply to the original MPPK/DS and are considered optimal attacks. In Table 4, the Constrained category offers much smaller sizes than original MPPK/DS for private key, public key, in addition to 20% smaller signature size, decreased from 160 to 128 bytes. We then compare optimized MPPK/DS Xsecure with NIST standardized algorithm Dilithium, Falcon, and SPHINCS+ for all three levels. In this case, Xsecure MPPK/DS with over 384 bits of entropy offers key sizes and signature size similar to RSA2048. For public key, MPPK/DS Xsecure has 256 bytes, comparing with 897 bytes at NIST level I, 1793 bytes at NIST level V for Falcon, and 1312 bytes at level I, 1952 bytes at level III, and 2592 bytes at level V for Dilithium, respectively. However, SPHINCS+ provides the smallest key sizes: 32 bytes for public key and 64 bytes for private key at level I, 48 bytes for public key and 96 bytes for private key at level III, and 64 bytes for public key and 128 bytes for private key, respectively, 4$\times$ smaller than MPPK/DS Xsecure for public key size.

In comparison with all NIST stadardized algorithms, MPPK/DS Xsecure offers the smallest signature size of 128 bytes with SHA256 as what the standardized algorithm used. Dilithium’s signature sizes are about 19$\times$ to 36$\times$ larger than MPPK/DS Xsecure’s and Falcon’s signature sizes 5$\times$ to 10$\times$ larger than MPPK/DS Xsecure’s. The most bigest signature sizes are from SPHINCS+, 60$\times$ to 390$\times$ bigger than MPPK/DS Xsecure’s. Overall, it can be seen that MPPK/DS Xsecure could be an optimal generic digital signature scheme for post-quantum era, applicable for any devices.

**Table 4:** Public key and signature sizes of the the optimized MPPK/DS scheme as well as MPPK/DS and the NIST PQC Round 3 finalists, with values given in bytes corresponding to various NIST security levels.

Signature scheme	Key Pair(PK,sk) (B)			Signature size (B)		
	I	III	V	I	III	V
MPPK/DS	(192, 64)	(288, 64)	(288, 80)	160	160	160
Constrained	(128, 64)	(128, 64)	(192, 80)	128	128	128
Xsecure	(256, 128)	(256, 128)	(256, 128)	128	128	128
Dilithium$^{\textrm{1}}$	(1312, n/a)	(1,952, n/a )	(2,592, n/a )	2,420	3,293	4,595
Falcon$^{\textrm{2}}$	(897, n/a )	–	(1,793, n/a )	666	–	1,280
SPHINCS+s	(32, 64)	(48, 96)	(64, 128)	7,856	16,224	29,792
SPHINCS+f	(32, 64)	(48, 96)	(64, 128)	17,088	35,664	49,856

$^{\textrm{1}}$ Dilithium does not provide primitive for NIST Level I, *dilithium3*.

was used for Level III, and *dilithium5* for Level V.

$^{\textrm{2}}$ For Falcon, *falcon512dyn* was measured for Level I, no primitive.

was measured for Level III, *falcon1024dyn* was measured for Level V.

The optimized MPPK/DS offers public key size $2m(n+\lambda )$ field elements of $\mathbb {F}_p$ and signature size 4*x* elements of $\mathbb {F}_p$ or message length in terms of *A*, *B*, *C*, *D*.

In comparison with the original MPPK/DS^6^, optimized MPPK/DS would offer better performances in key generations, signing, and verifying for all security levels are tabulated in Table 5. Performances for standardized algorithms are taken from their submission documents^13,14,18^. Overall, MPPK/DS Xsecure outperforms all the standardized algorithms for key generation, signing and verifying procedures. For key generation, MPPK/DS Xsecure takes about 26 K cycles for all security levels, the fastest algorithm comparing with Dilithium, Falcon, and SPHING+. The second fastest algorithm in key generation is Dilithium, then third is Falcon, and the slowest is SPHINCS+ which is four orders of magnitude slower than MPPK/DS Xsecure. For signing, the relative performance is the similar to the key generation procedure, MPPK/DS is the fastest and SPHINCS+ is the slowest, again four orders of magnitude slower than MPPK/DS. It is cearly seen from Table 5 that MPPK/DS signature verification is 4$\times$–6$\times$ faster than Dilithium, 10$\times$ faster than Falcon, and about 40$\times$ faster than SPHINCS+.

**Table 5:** Performance comparison of the optimized MPPK/DS Xsecure are tabulated for key generation, signing, and verifying against NIST standardized algorithms.

${\mathscr {O}}(\varphi (p)^{\lambda +2}2^xq^{-m})$	Level I	Level III	Level V
Xsecure	64.64.2.2.2	64.64.2.2.2	64.64.2.2.2
**Key Gen**			
MPPK/DS Xsecure	25,590	25,590	25,590
Dilithium	30,075	544,232	819,475
Falcon	19,872,000		63,135,000
SPHINCS$^+$-SHA-256-128s-simple	358,061,994	524,116,024	346,844,762
**Signing**			
MPPK/DS Xsecure	62,943	94,414	132,180
Dilithium	1,355,434	2,348,703	2,856,803
Falcon	3.362.418		6,865,774
SPHINCS$^+$-SHA-256-192s-simple	2,721,595,944	5,012,149,284	4,499,800,456
**Verifying**			
MPPK/DS Xsecure	71,823	108,125	143,646
Dilithium	327,362	522,267	871,609
Falcon	715,999		1,465,201
SPHINCS$^+$-SHA-256-256s-simple	2,712,044	4,333,066	6,060,438

All performance results are displayed in clock cycles. We choose SHA-256, SHA-384, and SHA-512 for MPPK/DS Xsecure security level I, III, and V respectively but only SHA-256 is selected for other standardized algorithms. That means, MPPK/DS Xsecure uses hash codes 32 bytes, 48 bytes, and 64 bytes for security level I, III, and V.

### Consideration of side-channel resistant implementation

Using optimized MPPK/DS to sign a message, we normally first calculate the univariate polynomials $a(x_0), b(x_0), c(x_0)$, and $d(x_0)$ and then perform the modular exponentiation evaluation with a randomly chosen base *g* from $\mathbb {F}_p$. These polynomial evaluations are associated with potential side-channel attacks proposed by Carlet and Prouf^38^. We propose to disassemble the polynomial evaluations into signing processes by combining polynomial evaluations and signing together as follows

$$\begin{aligned}{} & {} A = g^{a(x_0)} \ \text {mod p} = g^{a_0 + a_1x_0 + \dots + a_{\lambda } x_0^{\lambda }} = (g^{a_0}) (g^{a_1})^{x_0} \dots (g^{a_{\lambda }})^{x_0^{\lambda }} \ \text {mod p} \\{} & {} B = g^{b(x_0)} \ \text {mod p} = g^{b_0 + b_1x_0 + \dots + b_{\lambda } x_0^{\lambda }} = (g^{b_0}) (g^{b_1})^{x_0} \dots (g^{b_{\lambda }})^{x_0^{\lambda }} \ \text {mod p} \\{} & {} C = g^{c(x_0)} \ \text {mod p} = g^{c_0 + c_1x_0 + \dots + c_{\lambda } x_0^{\lambda }} = (g^{c_0}) (g^{c_1})^{x_0} \dots (g^{c_{\lambda }})^{x_0^{\lambda }} \ \text {mod p} \\{} & {} D = g^{d(x_0)} \ \text {mod p} = g^{d_0 + d_1x_0 + \dots + d_{\lambda } x_0^{\lambda }} = (g^{d_0}) (g^{d_1})^{x_0} \dots (g^{d_{\lambda }})^{x_0^{\lambda }} \ \text {mod p} \end{aligned}$$

with the random base *g*, the above implementation can avoid the side-channel analysis on the polynomials. Algorithm 1 illustrates the pseudo code of the implementation for signing process.
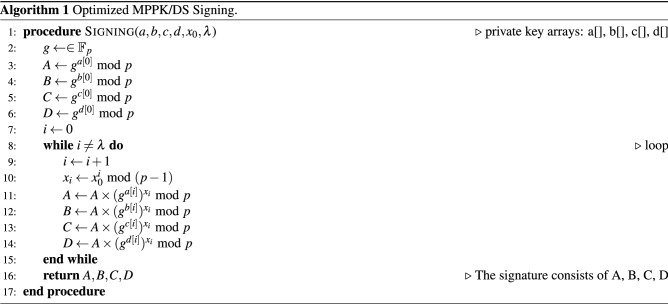


## Conclusion

In this work, we presented a new version of a novel quantum-safe digital signature algorithm called Multivariate Polynomial Public Key Digital Signature (MPPK/DS) introduced by Kuang, Perepechaenko, and Barbeau^6^. We presented an optimized version of the MPPK/DS schemes, with the significantly reduced public key and signature sizes, based on the newly identified optimal attack mechanisms. Security analysis given in the original version of MPPK/DS^6^ have been improved, and new more efficient attacks have been discovered. We include these attacks in this work. The optimized version of MPPK/DS does not include a fifth signature element *E*, and does not have any elements associated solely with the message variable $x_0$ compared to the original MPPK/DS. Moreover, we introduced two new private secret values $\alpha$ and $\beta$ used to obscure the public key polynomials ${\mathscr {P}}(\cdot )$ and ${\mathscr {Q}}(\cdot )$. We have provided a detailed description of the optimized MPPK/DS and illustrated it with a toy example. We also conducted an updated security analysis that includes most recent attacks that we have discovered. In particular, we describe some attacks that we have discovered on the Optimized MPPK/DS, as well as the attacks described in^6^ adapted to the optimized version. One of the biggest differences in the security analysis is the discovery of the optimal key recovery attack and a new improved spoofing attack with classical complexity of ${\mathscr {O}}(p^4).$ The optimized MPPK/DS offers two category configurations: Xsecure for maximum secure and constrained for resource limited devices. We also point out that the optimized version of MPPK/DS has smaller signature sizes, which now only include four signature elements. The preliminary performance comparisons demonstrated that optimized MPPK/DS outperforms all standardized algorithms for key generation, signing and verying procedures. We will report benchmarking performance for both categories separately.

We have also introduced side-channel resistant implementation of the optimized MPPK/DS and provide a pseudo-code for the implementation. Overall, optimized MPPK/DS is a great improvement of the original MPPK/DS scheme without any compromise to the security or the construction of the scheme. We will report on the performance of the optimized MPPK/DS separately, and will consider it as a standalone algorithm as well as in comparison with the original MPPK/DS and NIST standardization candidates to provide a full idea of the possible use cases of the optimized MPPK/DS.

We are also currently working on modifying the MPPK/DS algorithm to resist a recent attack proposed by Guo^40^.

## Data Availability

All data generated or analyzed during this study is included in this published article.
